# A knock down strategy for rapid, generic, and versatile modelling of muscular dystrophies in 3D-tissue-engineered-skeletal muscle

**DOI:** 10.1186/s13395-024-00335-5

**Published:** 2024-02-22

**Authors:** Stijn L. M. in ‘t Groen, Marnix Franken, Theresa Bock, Marcus Krüger, Jessica C. de Greef, W. W. M. Pim Pijnappel

**Affiliations:** 1 Department of Clinical Genetics, Erasmus University Medical Center, Rotterdam, 3015 GE The Netherlands; 2 Department of Pediatrics, Erasmus University Medical Center, Rotterdam, 3015 GE The Netherlands; 3 Center for Lysosomal and Metabolic Diseases, Erasmus Medical Center, Rotterdam, 3015 GE The Netherlands; 4 Institute of Genetics and Cologne Excellence Cluster on Cellular Stress Responses in Aging-Associated Diseases (CECAD), University of Cologne, Cologne, Germany; 5 Center for Molecular Medicine, University of Cologne, Cologne, Germany; 6 Department of Human Genetics, Leiden University Medical Center, Leiden, 2333 ZA Netherlands

**Keywords:** Duchenne muscular dystrophy, LGMD2A, Disease modelling, Gene knockdown, 3D-organ-on-a chip, Skeletal muscle-on-a-chip, Tissue engineering, Dystrophin, Calpain-3

## Abstract

**Background:**

Human iPSC-derived 3D-tissue-engineered-skeletal muscles (3D-TESMs) offer advanced technology for disease modelling. However, due to the inherent genetic heterogeneity among human individuals, it is often difficult to distinguish disease-related readouts from random variability. The generation of genetically matched isogenic controls using gene editing can reduce variability, but the generation of isogenic hiPSC-derived 3D-TESMs can take up to 6 months, thereby reducing throughput.

**Methods:**

Here, by combining 3D-TESM and shRNA technologies, we developed a disease modelling strategy to induce distinct genetic deficiencies in a single hiPSC-derived myogenic progenitor cell line within 1 week.

**Results:**

As proof of principle, we recapitulated disease-associated pathology of Duchenne muscular dystrophy and limb-girdle muscular dystrophy type 2A caused by loss of function of DMD and CAPN3, respectively. shRNA-mediated knock down of DMD or CAPN3 induced a loss of contractile function, disruption of tissue architecture, and disease-specific proteomes. Pathology in DMD-deficient 3D-TESMs was partially rescued by a candidate gene therapy treatment using micro-dystrophin, with similar efficacy compared to animal models.

**Conclusions:**

These results show that isogenic shRNA-based humanized 3D-TESM models provide a fast, cheap, and efficient tool to model muscular dystrophies and are useful for the preclinical evaluation of novel therapies.

**Supplementary Information:**

The online version contains supplementary material available at 10.1186/s13395-024-00335-5.

## Introduction

Neuromuscular disorders comprise a broad range of diseases that result in skeletal muscle weakness. Muscular dystrophies represent a class of neuromuscular disorders that directly affects skeletal muscle, with severe impact on patients, including immobilization, respiratory insufficiency, reduced quality of life, and shorter life expectancy [1]. There are currently very few treatment options available for neuromuscular disorders, with some exceptions including enzyme therapy for Pompe disease and gene therapy, antisense oligonucleotides, and splice modulators for spinal muscular atrophy [2–4]. One reason for the limited number of available treatments is the lack of suitable humanized disease models that are able to mimic disease pathology and that allow the functional evaluation of novel therapeutic interventions [5, 6]. Transgenic mouse models have been generated for many muscular dystrophies, but these often have mild phenotypes and may not (fully) represent disease mechanisms that operate in human patients due to species-specific differences [7]. It has therefore been difficult to translate results obtained in mouse models to human patients, resulting in a very low success rate of candidate drugs in clinical trials [7]. In vitro systems for skeletal muscle include 2D cultures of differentiated human skeletal muscle cells, which provide an easy approach for the identification of therapeutic targets. However, 2D cultures lack the structural organization of skeletal muscle tissue and are often not suitable for functional assessments such as contractile force [8, 9].

The introduction of new models based on organoids, microtissues, and organ-on-a-chip platforms has allowed for more complexity of in vitro human disease models [8–10]. For skeletal muscle, this has resulted in the development of 3D-tissue-engineered-skeletal muscles (3D-TESMs) that aim to recapitulate the native architecture of skeletal muscle and that can provide a functional readout through contractility measurements [8, 9]. Neuromuscular disorders can be modeled with 3D-TESMs using primary or hiPSC-derived muscle cells. It is becoming clear that there is a considerable heterogeneity among human individuals due to differences in genetic backgrounds, resulting in large variation between samples and difficulty to distinguish between natural variation and disease phenotypes [10, 11]. This variability can be reduced with the generation of isogenic pairs, in which healthy and diseased models are generated with identical genetic backgrounds, obtained either using gene editing or using patients who are mosaic for the gene defect. The generation of isogenic disease models is laborious and time-consuming, thereby limiting throughput of disease modelling applications [11, 12]. In the case of hiPSC-derived 3D-TESMs, the time required to generate these involves approximately 2 months for hiPSC generation, 2 months for gene editing to generate isogenic controls, and 2 months for the generation and expansion of myogenic progenitors [13, 14].

Here, we developed a method for rapidly modelling of neuromuscular diseases employing isogenic models using a highly efficient shRNA-based knock down strategy in 3D-TESMs [15]. As a proof of principle, we induced genetic deficiencies for *DMD* (Duchenne muscular dystrophy, OMIM#310200) and *CAPN3* (limb-girdle muscular dystrophy type 2A (LGMD2A), OMIM#253600) in healthy hiPSC-based 3D-TESMs. This resulted in a rapid knock down of DMD and CAPN3 proteins, which in both cases caused loss of contractile function within a week. Mass spectrometry analysis showed disease-specific proteomic signatures upon knock down that reflected proteomic changes that have been reported in patient-derived skeletal muscle biopsies. Expression of *micro-dystrophin*, a potential novel gene therapy product for Duchenne muscular dystrophy, in 3D-TESMs partially rescued the dystrophic phenotype caused by *DMD* knock down [16, 17]. These results show that the shRNA-mediated knock down approach in 3D-TESMs provides a fast and efficient tool to model muscular dystrophies for the analysis of disease mechanisms and novel treatment options.

## Methods

### Myogenic progenitor culture and 2D differentiation

hiPSC-derived MPCs were generated from healthy iPSCs using a transgene-free protocol and expanded as described previously (Table S1) [13]. In brief, MPCs were expanded on ECM (1:200)-coated dished in proliferation medium consisting of high glucose DMEM supplemented with 10% FBS, 1% penicillin/streptomycin/glutamine (p/s/g), and 100 ng/ml FGF2 (PrepoTech). Myogenic differentiation into myotubes was induced with differentiation medium consisting of high glucose DMEM, 1% knockout serum replacement, 1% ITS-X, and 1% p/s/g.

### Generation of hiPSC-derived myogenic progenitors 3D-TESM

3D-TESMs were generated in an Ecoflex Replica mold using a 15-µl hydrogel-cell mixture containing 2 mg/ml fibrinogen (Sigma-Aldrich), 20% Matrigel growth factor reduced (±10 mg/ml, Corning), 240,000 MPCs, and 0.8 units/ml bovine thrombin (Sigma-Aldrich) [15]. For the delivery of lentiviral particles, viral concentrates were transferred to the hydrogel-cell mixture before pipetting the mixture into the PDMS chamber or to the culture medium. The 3D-TESMs were incubated for 20 min at 37 °C before the addition of proliferation medium supplemented with 1.5 mg/ml 6-aminocaproic acid (6-ACA) (Sigma-Aldrich) and switched to differentiation medium supplemented with 2 mg/ml 6-ACA after 48 h. 3D-TESMs were cultured on a 65-rpm shaking platform at 37 °C/5% CO2, and half of the differentiation medium was refreshed every 48 h.

### Force measurements

Before stimulation, 3D-TESMs were left at room temperature for 10 min to prevent spontaneous contractions. Electrical stimulation was performed at a frequency of 1 Hz (twitch) or 20 Hz (tetanus) with 2.5 V using carbon plate electrodes wired to an Arduino Uno Rev3 at both ends of the chamber. The displacement of the pillars was recorded with optical imaging and analyzed with ImageJ. The position at the pillar was determined with images from the side taken immediately after stimulation. Forces were calculated in N with the formula *N* = (6E πr^4)/(4a^2(3L-a))δ using a previously determined PDMS stiffness of 1.8067 MPa.

### RNA isolation and cDNA synthesis

3D-TESMs were dissociated using 700-µl QIAzol lysis buffer (Qiagen) and a TissueRuptor (Qiagen) for ~10 s. Next, 140-µl chloroform was added and incubated for 5 min at room temperature. Lysates were thereafter centrifuged at 10,000 rpm, and the aqueous layer was isolated and mixed with 525-µl 100% ethanol. Finally, the RNeasy Micro Kit (Qiagen) including a 15-min DNase incubation was used to purify RNA and converted into cDNA using the iscript cDNA synthesis kit (Qiagen) following the manufacturer’s manuals. For RT-qPCR, cDNA was diluted 10× and performed as described previously [13]. Primers are shown in Table S1.

### Whole-mount immunofluorescence staining

Immunofluorescent staining of intact 3D-TESMs was performed as described previously [15] using anti-titin (DSHB 1:50), anti-mouse IgM (Invitrogen 1:500), and Hoechst nuclear staining (1:15,000 final dilution). After staining, TESMs were stored in 80% glycerol and imaged using a Leica TCS SP5 confocal microscope.

### 3D-TESM dissociation and flow cytometry

3D-TESMs were enzymatically dissociated for 1 h using 10× TrypLE (Gibco). Next, the cell suspension was passed through a 40-µm cell strainer (Corning) and centrifuged for 5 min at 1000 rpm. Cells were then resuspended in 2% PFA, incubated for 15 min at room temperature, centrifuged for 5 min at 1000 rpm, and resuspended in 200-µl PBS. Flow cytometry of the fixed cells was performed using a BD LSR Fortessa, measuring the GFP signal in 50,000 cells. The data were analyzed and visualized using FlowJo software.

### Lentivirus production and titering

The *H2B-GFP* lentiviral vector was generated by cloning *H2B-GFP* into a pCCL lentiviral vector using the BamHI and SpeI sites (New England Biolabs). shRNAs were obtained as bacterial stocks from the MISSION shRNA library. Viral concentrates were generated as described in [18, 19]. Lentiviral titers were determined by transducing the lentiviral particles onto 240,000 MPCs in a dilution curve. Four days after transduction, the lentiviral titer was determined using qPCR to check the vector copy numbers with primers specific for the integrated lentiviral construct (Table S2).

### Vector copy number quantification

Genomic DNA was isolated from 2D cultured MPCs at 24 h after lentiviral transduction. Vector copy number (VCN) was analyzed by qPCR, using primers specific for the integrated lentiviral construct (Table S2). Absolute quantification of the VCN was performed using albumin as standard and a previously generated stable “1 copy per genome” cell line [19].

### Proteomic analysis

The proteomic analysis is described in full in the Supplementary materials. In short, proteome samples were analyzed using a Q-Exactive™ Plus Hybrid Quadrupole-Orbitrap™ Mass Spectrometer (Thermo Fisher). Demultiplexing of the MS raw data was achieved by ProteoWizard (3.0.21218) and analyzed using DIA-NN analysis software (1.8.1) [20]. Statistical analysis was performed using Perseus (1.6.15.0). FDR cutoff was set to ≤ 0.05 compared to the respective control in ≥ 2 timepoints, and GO pathway enrichment was analyzed using the protein string database. Graphical visualization was done in GraphPad Prism and InstantClue [21]. The mass spectrometry proteomics data have been deposited to the ProteomeXchange Consortium via the PRIDE partner repository with the dataset identifier PXD042227.

## Results

### Hydrogel delivery of lentiviral particles

An *H2B-GFP* lentivirus was generated to assess the efficiency of lentiviral delivery to 3D-tissue-engineered-skeletal muscles (3D-TESMs). We first tested whether lentiviral transduction and *H2B-GFP* overexpression might affect myogenic differentiation. To this end, myogenic progenitor cells (MPCs) grown in 2D were transduced by the addition of virus to the medium [13]. Analysis was performed 48 h after transduction and showed that 88% of nuclei were GFP positive (Fig. S1). Next, myogenic differentiation was induced through serum deprivation, and immunofluorescent staining with a myosin heavy chain (MYH) antibody was used to detect terminally differentiated MYH-positive multinucleated myofibers. We observed similar fusion indexes between the transduced and untransduced MPCs (79% and 84%, respectively, Fig. S1). GFP-positive nuclei were found to be present throughout the terminally differentiated myotubes after lentiviral targeting with a similar nuclear distribution as untransduced myotubes. This indicates that the MPCs retained their myogenic potential upon lentiviral transduction with H2B-GFP.

**Fig. 1:**
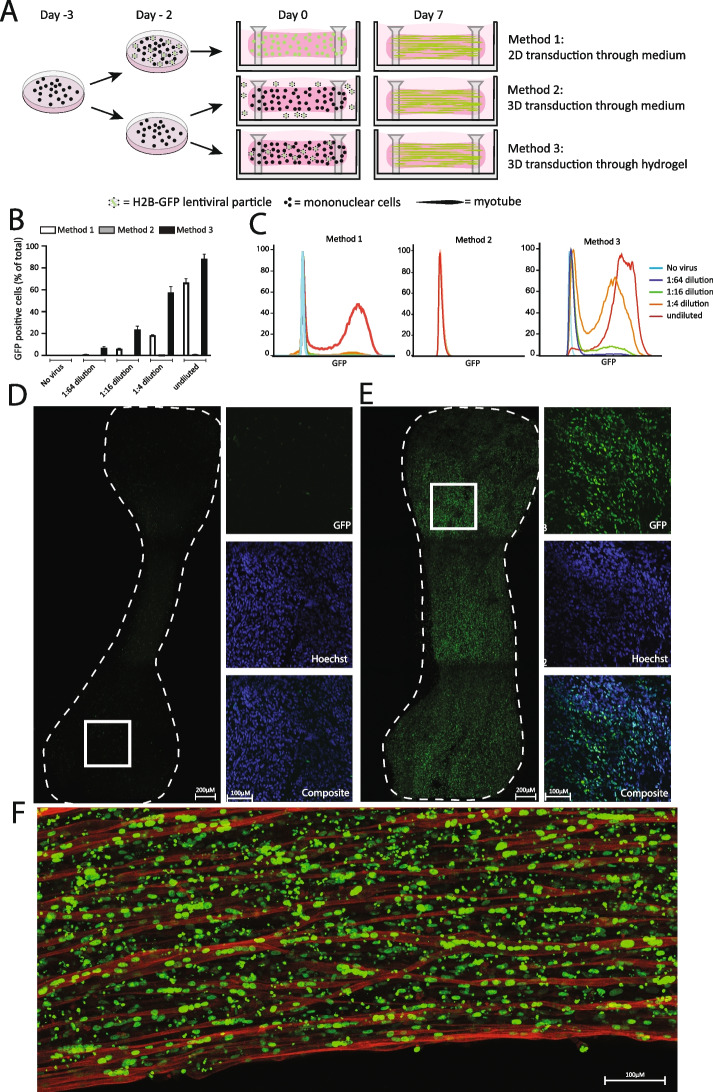
Efficient lentiviral transduction of 3D-tissue-engineered-skeletal muscle. **A** Experimental approach. Method 1: cells were transduced in 2D, before the generation of 3D-TESMs. Method 2: 3D-TESMs were generated from untransduced cells and were then transduced with lentiviral particles delivered through the cell culture medium. Method 3: 3D-TESMs were generated from untransduced cells and were then transduced with lentiviral particles delivered through the hydrogel during 3D-TESM formation. An H2B-GFP *Lentivirus* was used to assess the efficacy of the delivery method. **B** FACS analysis of the number of GFP-positive cells as a percentage of the whole population for the three delivery methods. Four H2B-GFP lentiviral concentrations were tested per method. Data are derived from three independent 3D-TESMs and expressed as mean ± SD. **C** Representative FACS plots from **B**. **D** Whole-mount immunofluorescent analysis of 3D-TESMs showing GFP 48 h after transduction with undiluted *H2B-GFP* virus using Method 2. Zoomed-in region of a single Z-stack is shown on the right. Nuclei were stained with Hoechst (in blue). **E** as **D** but for Method 3. **F** Sagittal Z-stack of 3D-TESMs transduced with Method 3, after 7 days of myogenic induction, stained with an anti-titin antibody (red)

Next, we evaluated different lentiviral delivery methods for transducing 3D-TESMs: via the medium of MPCs grown in 2D, followed by 3D-TESM generation (Method 1) and via the medium of preformed 3D-TESMs (Method 2): or by mixing lentiviruses with MPCs and the hydrogel during 3D-TESM formation (Method 3) (Fig. 1A). Lentiviral transduction was tested at 1:4 serial dilutions, and after 48 h, the percentage of GFP-positive cells was determined using flow cytometry. A targeting efficiency of ± 70% was obtained when using method 1 with the undiluted viral titer (Fig. 1B–C). Transduction via the culture medium in 3D-TESMs (Fig. 1, Method 2) resulted in a very low efficiency of < 1% at the highest titer. In contrast, transduction via the hydrogel (Fig. 1, Method 3) resulted in enhanced efficiency compared to Method 1 of up to 88% at the highest titer (Fig. 1B–C). Serial dilutions of the lentivirus resulted in concomitant lower transduction efficiencies. Confocal microscopy confirmed these results (Fig. 1D). Only a minor fraction of GFP-positive nuclei were detected in the 3D-TESMs that were transduced via Method 2, in which GFP-positive nuclei were confined to the outer layer of the 3D-TESMs, indicating that lentiviral particles were unable to penetrate into the interior of the tissue using this delivery method. Analysis of the 3D-TESMs that were transduced via Method 3 showed that almost all nuclei were GFP positive (Fig. 1D). GFP-positive nuclei were found to be equally distributed throughout the 3D-TESMs, indicating that in this delivery method, lentiviral particles were able to target cells of both the inner and outer layers of the 3D-TESMs. As a final confirmation, myogenic differentiation was induced in the 3D-TESMs that were transduced with Method 3 for a 7-day period. Analysis of these 3D-TESMs, using titin (TTN) as a marker to identify terminally differentiated myofibers, showed an abundance of GFP-positive nuclei in between and within differentiated myofibers (Fig. 1E), indicating that transduced MPCs were able to undergo myogenic differentiation in 3D-TESMs.

### Generation of 3D-TESM disease models through shRNA-mediated knockdown

To apply lentiviral transduction for disease modelling using 3D-TESMs, lentiviruses expressing 2–3 shRNA targeting sequences per gene for d*ystrophin* (*DMD*), c*alpain-3* (*CAPN3*), or *myostatin* (*MSTN*) were tested individually (Table 1). Optimal viral titers required for knock down were determined by transducing MPCs in 2D using serial dilutions for each target sequence, followed by RT-qPCR analysis. 3D-TESMs were then generated and transduced using hydrogel delivery of shRNA expressing lentiviruses (Method 3). Myogenic differentiation was induced for a period of 7 days, and 3D-TESM tissues were analyzed for force-generating capacity in response to electrical stimulation, expression of the targeted gene, and tissue morphology using whole-mount immunofluorescence of TTN (Figs. S2–S4). The 3D-TESMs that were found to have a > 50% reduction of *CAPN3* (shRNA #2 and #3) or *DMD* (shRNA #1 and #3) expression after shRNA transduction showed a reduced or a complete lack of contractile force and a reduction of aligned TTN-positive myofibers (Figs. S2 and S3). No significant differences in morphology or contractile force were found between the 2 *MSTN* knock down shRNAs and non-targeting control 3D-TESMs, while both targeting shRNAs caused a significant reduction in the expression of *MSTN* (Fig. S4).

**Table 1:** Viral constructs used in this study

Lentiviral construct	Insert or shRNA target sequence	Experiment	Vector origin
H2B-GFP	*H2B-GFP*	Figures 1 and S1	
Non-targeting shRNA	AACAAGATGAAGAGCACCAACT	Figures 2, 3, 4 and S2–S9	MISSION-shRNA library; SHC002
*CAPN3-*targeting shRNA #1	CCGCAACTTCCCAGATACTTT	Figure S2	MISSION-shRNA library; TRCN0000003493
*CAPN3-*targeting shRNA #2	CGGAGTGAAAGAGAAGACATT	Figure S2	MISSION-shRNA library; TRCN0000003494
*CAPN3-*targeting shRNA #3	GCTCCTGCTTACCTTGCTCTA	Figures 2, 3, S2, S5–S9	MISSION-shRNA library; TRCN0000003495
*MSTN-*targeting shRNA #1	CCCACAAAGATGTCTCCAATT	Figure S3	MISSION-shRNA library; TRCN0000059138
*MSTN-*targeting shRNA #2	CCTGAATCCAACTTAGGCATT	Figures 2, 3, S3, S5–S9	MISSION-shRNA library; TRCN0000059140
*DMD-*targeting shRNA #2	CCAGCATTACTGCCAAAGTTT	Figure S4	MISSION-shRNA library; TRCN0000053245
*DMD-*targeting shRNA #3	CCAGTCTTTAGCTGACCTGAA	Figures 2, 3, S4, S5–S9	MISSION-shRNA library; TRCN0000053246
*DMD-*targeting shRNA #4	CCCTAGTTCAAGAGGAAGAAA	Figure 4	MISSION-shRNA library; TRCN0000053243
Micro-dystrophin	*Micro-dystrophin*	Figure 4	Chamberlain lab; Addgene plasmid #26810
GFP	*GFP*	Figure 4	

**Fig. 2:**
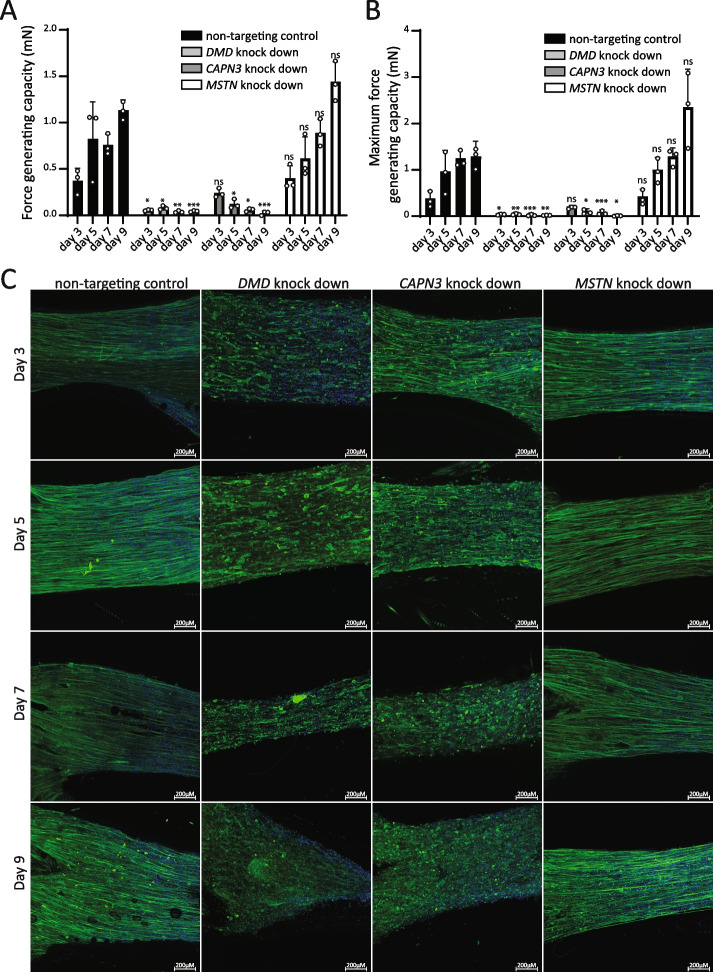
shRNA-mediated knock down of *DMD*, CAPN3, and *MSTN* in 3D-tissue-engineered-skeletal muscle. **A** Effect of shRNA-mediated knock down on force-generating capacity (twitch force) in 3D-TESMs. 3D-TESMs were transduced (using Method 3) with shRNAs targeting *DMD*, *CAPN3*, or *MSTN*. Force-generating capacity was measured in response to stimulation at 1 Hz. Data are represented as mean ± SD derived from three independent 3D-TESMs at each timepoint (12 3D-TESMs total) for all conditions. Statistical significance is indicated for all conditions, compared to the corresponding non-targeted control. *p < 0.05, **p < 0.01, ***p < 0.001. **B** as **A** but now showing the maximum force-generating capacity in response to tetanic stimulation (20 Hz). **C** Whole-mount immunofluorescent stainings of all experimental conditions from **A** and **B**. Green, anti-titin antibody. Blue, nuclei stained with Hoechst

Next, we performed a time course experiment to characterize the effects of *DMD*, *CAPN3*, and *MSTN* knock downs in 3D-TESMs in more detail. Using the best-performing shRNA construct from Figs. S2–S4 for each target gene (Table 1), we analyzed myofiber diameter (Fig. S6A), myofiber alignment (Fig. S6B), the force generating capacity of 3D-TESMs in response to twitch stimulation (Fig. 2A; “force-generating capacity”), and tetanic stimulation (Fig. 2B; “maximum force-generating capacity”) after 3, 5, 7, and 9 days of 3D-TESM formation. 3D-TESMs that were transduced with the non-targeting shRNA generated contractile forces of ~0.4 mN already at 3 days of differentiation and showed a gradual increase in force-generating capacity to a maximum of ~1.2 mN at day 9. Parallel-oriented TTN-positive myofibers were observed from day 3 of differentiation onwards (Fig. 2C). A strong phenotype was observed for the knock down of *DMD* in 3D-TESMs. Contractile forces were close to zero starting from day 3 of differentiation onwards (Fig. 2A–B). This was paralleled by morphology: from day 3 of differentiation onwards, TTN-positive myofibers were shortened, they lacked cross-striation, and the 3D-TESMs appeared disorganized, lacking fiber alignment (Figs. 2C and S6B).

Knock down of *CAPN3* in 3D-TESMs resulted in contractile forces that were similar to those generated in the non-targeting controls at day 3 of differentiation (Fig. 2A–B). From day 5 of differentiation onwards, *CAPN3* knock down caused reduced contractility, ultimately resulting in a complete absence of contractile response on day 9 of differentiation. Also in this case did the morphology parallel the force-generating capacity: aligned and cross-striated myofibers were present at day 3 of differentiation, but from day 5 onwards, fiber organization appeared irregular and disrupted, which was accompanied by a progressive increase in the presence of TTN-positive spherical structures (Figs. 2C and S6). We hypothesize that these structures represented partially detached myofibers, since their increase appeared to correspond with the loss of myofibers. At day 9 of differentiation, striated myofibers were no longer present.

Knock down of *MSTN* failed to cause significant effects on force-generating capacity, although a nonsignificant increase in twitch and tetanic force was observed at day 9 of differentiation (Fig. 2A–B). The morphology and organization of the myofibers appeared unchanged (Fig. 2C). Lentiviral transduction resulted in a vector copy number (VCN) of 2–6 (Fig. S5A, B), and did not affect fusion index in myotubes grown in 2D (Fig. S5C, D). This suggest that the knock downs did not interfere with formation of myotubes, and that the observed phenotypes resulted from pathology-induced downstream of myogenic fusion. For all knock downs, tissue diameter appeared similar (Fig. S7). We conclude that knock down of *DMD* and *CAPN3*, but not *MSTN*, caused a severe reduction of contractile force in 3D-TESMs from days 3 to 5 until day 9, the last day tested, which was accompanied by disruption of myofiber morphology and organization.

### Proteomic analysis of shRNA-mediated knock downs in 3D-TESMs

**Fig. 3:**
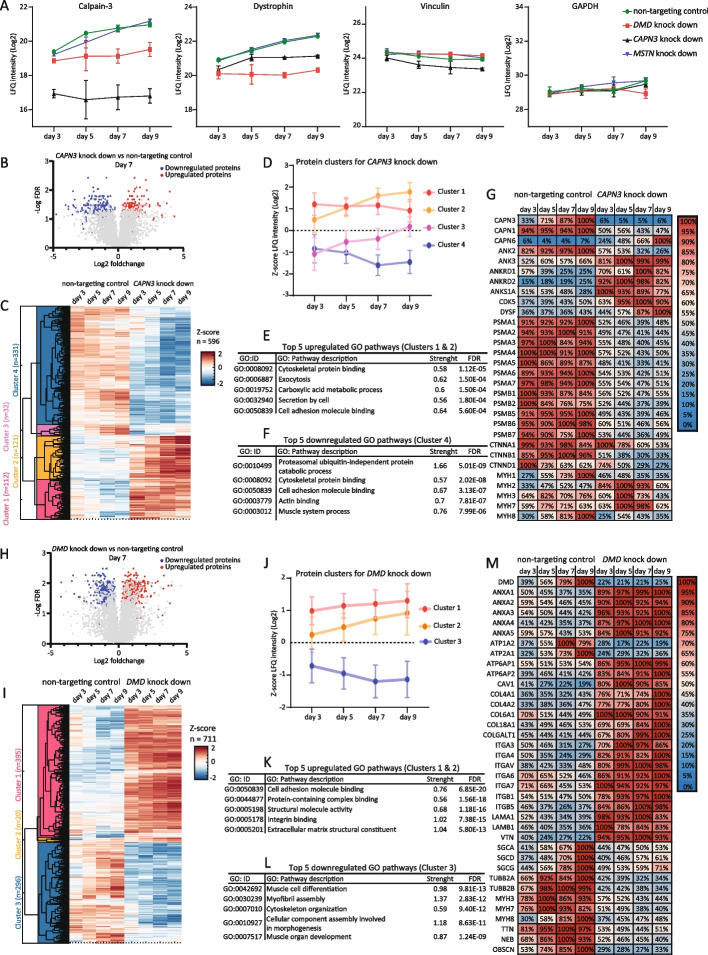
Proteomic analysis of *DMD* and *CAPN3* knock downs in 3D-tissue-engineered-skeletal muscle. **A** Effect of knock down on Calpain-3 (CAPN3) and dystrophin protein levels as a function of time in 3D-TESMs. For reference, the abundances of vinculin and GAPDH are shown. Data are expressed as log2 LFQ values and represented as means ± SD derived from three independent 3D-TESMs at each timepoint. **B** Volcano plot of the *CAPN3* knock down compared to the non-targeting control at day 7. Proteins that were significantly up and downregulated are indicated in red and blue, respectively. Average protein LFQ values were derived from three independent 3D-TESMs from each timepoint. Proteins were considered significant when FDR was < 0.05 in ≥ 2 timepoints. **C** Supervised clustering of the *CAPN3* knock down. Clusters with similar expression changes over time are indicated. Clustering was based on the Z-scores of the LFQ values of significant proteins in the *CAPN3* knock down relative to the non-targeting control. *n* = total number of proteins per cluster. **D** Z-scores of each protein cluster from **C**. **E** Top 5 enriched GO pathways in the upregulated protein clusters of the *CAPN3* knock down. **F** Top 5 enriched GO pathways in the downregulated protein clusters of the *CAPN3* knock down. **G** Heatmap of proteins found significantly altered in the *CAPN3* knock down relative to the non-targeting control. Colors and numerical values represent the mean LFQ as a percentage of the most enriched condition. **H**–**M** as **B**–**G** but for the knock down of *DMD*

We performed mass spectrometry of 3D-TESMs to validate knock down of targeted genes at the protein level and to test whether disease-specific proteomic profiles were obtained. Relative levels of Calpain-3 and dystrophin were reduced at all timepoints of knock downs of *CAPN3* and *DMD*, respectively, compared to non-targeting control or *MSTN* knock downs (Fig. 3A). This effect was most pronounced at the latest timepoint, resulting in an 18-fold reduction of Calpain-3 following *CAPN3* knock down and a 5-fold reduction of dystrophin in the *DMD* knock down. Levels of control proteins vinculin and GAPDH were similar for all knock downs at all time points. Myostatin levels were undetectable, precluding their analysis.

To analyze disease-specific protein signatures, we performed unsupervised clustering of all significant proteins, based on the Z-scores for each protein normalized for the non-targeting control for each respective timepoint. Expression of a total of 596 proteins was significantly changed (*FDR* < 0.05 in ≥ 2 timepoints) for the knock down of *CAPN3* (Fig. 3B). Four distinct protein clusters were identified based on their expression from d3 to d9 of culture, resulting in two clusters with upregulated (cluster 1 and 2) and one cluster with downregulated proteins (cluster 4) compared to the untargeted control (Fig. 3C–D). Cluster 3 contained only 32 proteins and could not be classified as up or downregulated and was therefore omitted from GO enrichment analysis. GO enrichment analysis of clusters 1, 2, and 4 showed enrichment of pathways involved in cell adhesion and cytoskeletal binding in both up- and downregulated protein clusters. In the downregulated protein cluster 4, enrichment of proteins associated with the proteasomal complex (PSMA3, PSMA5, PSMB3, PSMB4, PSB2, PSB3) and several pathways involved in skeletal muscle contractility was observed (Fig. 3D–F). These results parallel studies performed using muscle biopsies or primary cell lines from LGMD2A patients describing downregulation of the 26S proteasome and dysregulation of cytoskeletal proteins due to defects in the proteolytic function of Calpain-3 in these patients [22, 23].

Knock down of *DMD* in 3D-TESMs resulted in 711 significantly altered proteins that could be divided in three protein clusters: clusters 1 and 2 with upregulated proteins and cluster 3 with downregulated proteins compared to the non-targeting control (Fig. 3H–J). GO enrichment analysis showed that the downregulated cluster was enriched for proteins involved in the development and function of skeletal muscle tissues (MYHs, NEB, OBSCN, ATP1A2, ATP2A1) (Fig. 3I). The upregulated protein clusters were found to be enriched for proteins involved in cell-matrix attachment and ECM proteins, including ANXA1, COL4A1, COL4A2, COL8A1, LAMA1, ITGA3, and VTN (Fig. 3J–K). These results are in agreement with results obtained in the *mdx* mouse model and in primary muscle biopsies from human patients, whereby fibrosis is one of the main pathological features of Duchenne muscular dystrophy [24].

The proteomic analysis also offered an opportunity to analyze proteomic changes that occur during human muscle development in vitro. To this end, we analyzed the non-targeting control during 3D-TESM development and found 1282 proteins that were significantly altered between at least 2 timepoints of development. These proteins could be classified within one of five distinct protein clusters based on their expression pattern (Fig. S8A–B). Quality controls are shown in Fig. S9. Gene Ontology (GO) enrichment analysis was performed for the two main protein clusters, cluster 1 (*n* = 408; upregulated during development) and cluster 5 (*n* = 519; downregulated during development) (Fig. S8A–B). Cluster 1 was predominantly associated with cellular respiration, muscle contraction, and glucose metabolism, while cluster 5 contained proteins involved in the regulation of mRNA processing and stability (Fig. S8D–E). The developmental stage of skeletal muscle is characterized by the expression of distinct myosin heavy chain (MYH) isoforms. The embryonic isoform (MYH3) was the most abundant isoform at all timepoints. During the development of 3D-TESMs, there was a gradual increase of MYH1 expression (associated with both fetal and adult fibers), MYH4 (postnatal and adult fibers), and MYH8 (embryonic and fetal fibers) from day 3 to day 9 (Fig. S8E). This reflects a gradual maturation from the embryonic into the neonatal stage that occurs during the normal development of skeletal muscle [25]. In addition, at day 9, the highest abundance of sarcomeric proteins including TTN, NEB, ACTN3, and OBSCN were found, while proteins associated with the initial stages of myogenesis (e.g., MYOG, MYOF, ACTN4) showed reduced expression (Fig. S8F). This indicates that at 9 days, the hiPSC-derived 3D-TESMs had completed the initial stages of skeletal muscle development (e.g., myoblast fusion and sarcomere formation) and had entered the process of skeletal muscle maturation to a neonatal developmental stage, similar to other 3D-TESM models published to date [26–29].

### Micro-dystrophin rescues the DMD phenotype

**Fig. 4:**
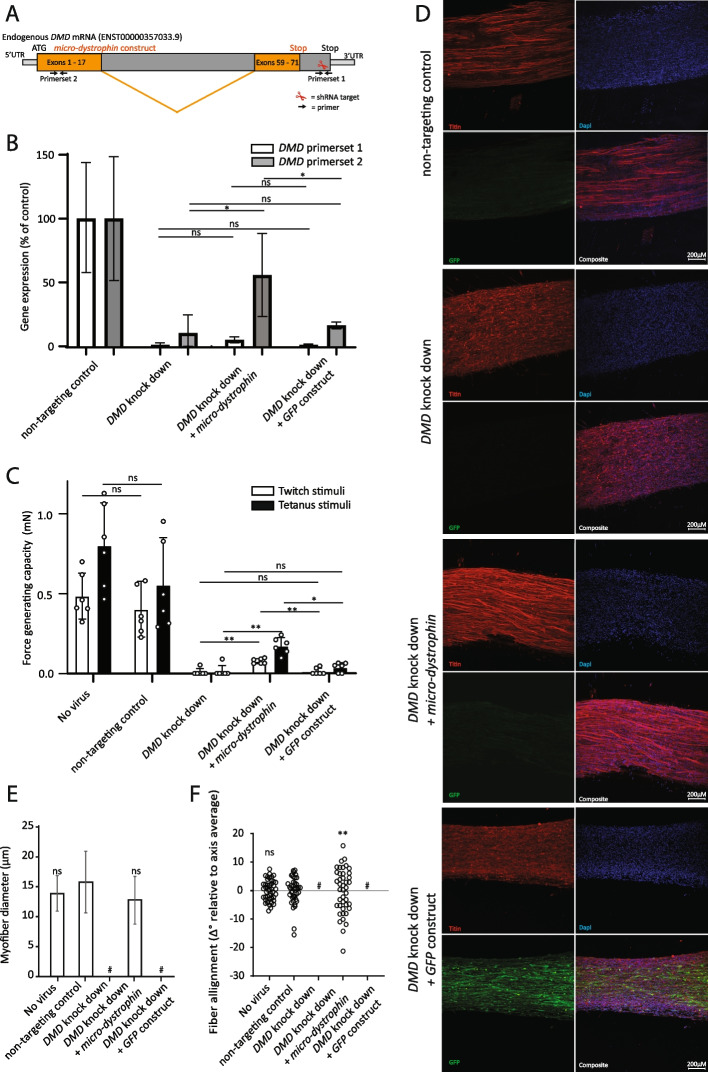
Rescue of *DMD* knock down by *micro-dystrophin* in 3D-tissue-engineered-skeletal muscle. **A** Cartoon of endogenous *DMD* and *micro-dystrophin* mRNA. The subset of *DMD* exons used to generate *micro-dystrophin* is indicated in orange. RT-qPCR primers that are specific for endogenous *DMD* (primer set 1) or that recognize both endogenous *DMD* and *micro-dystrophin* (primer set 2) are indicated. **B** RT-qPCR analysis of endogenous *DMD* and *micro-dystrophin* mRNAs. **C** Force-generating capacity (twitch stimulation) and maximum force-generating capacity (tetanic stimulation) of 3D-TESMs after 7 days of myogenesis. Data are represented as mean ± SD derived from six independent 3D-TESMs. **D** Representative images of whole-mount immunofluorescent stainings of experimental conditions from **B** and **C**. Red, anti-titin antibody. Green, GFP. Blue, nuclei stained with Hoechst. **E** Myofiber diameter of 3D-TESMs (*n* = 40–60 myofibers for each condition). **F** Myofiber alignment of 3D-TESMs. Myofiber alignment was measured using *Δ* of the angle of each myofiber relative to the perpendicular axis of the 3D-TESM (*n* = 40–60 fibers per condition). Statistical significance is indicated relative to the *DMD* knock down (**B** and **C**) or non-targeting control (**E** and **F**). For **F**, significance was calculated using the distribution of the variance. ns, not significant, *p < 0.05, **p < 0.01. #Indicates the conditions where the analyses were prevented by the poor quality of the 3D-TESM tissues

We utilized the 3D-TESM system to evaluate the efficacy of *micro-dystrophin*, a truncated version of *DMD* that is currently under evaluation as gene therapy in clinical trials, to rescue the Duchenne phenotype in vitro. Since the *micro-dystrophin* and *DMD* cDNAs share most of their coding regions, rendering *micro-dystrophin* susceptible to the *DMD-*targeting shRNAs, we generated *DMD*-targeting shRNA#4 to specifically target a region that was only present in the endogenous *DMD* gene but not in *micro-dystrophin* (Fig. 4A). Using RT-qPCR with primerset 1 that was designed to only amplify endogenous *DMD* transcripts, a ~90% reduction of *DMD* expression was obtained upon knock down using *DMD*-targeting shRNA #4 (Fig. 4B). Combining *DMD*-targeting shRNA #4 with a second lentiviral expression construct, expressing either *micro-dystrophin* or *GFP*, did not affect expression of endogenous *DMD*. RT-qPCR with primerset 2 was designed to amplify both *micro-dystrophin* and endogenous *DMD* transcripts showed that *micro-dystrophin* was expressed upon inclusion of the lentiviral expression construct. Similar to the results obtained with the *DMD*-targeting shRNAs #1–3 (Figs. 2 and 3), *DMD*-targeting shRNA #4 caused a strong reduction of contractile force in 3D-TESMs (Fig. 4C). Co-expression of *micro-dystrophin* in DMD shRNA #4 targeted 3D-TESMs resulted in a significant increase in both the force-generating capacity (twitch) and the maximum force-generating capacity (tetanus). However, the *micro-dystrophin*-induced rescue of contractile force in DMD 3D-TESMs was only partial, reaching 20% (twitch) and 30% (tetanus) of forces reached by the non-targeting control. As a control, co-expression of GFP in *DMD* shRNA #4-targeted 3D-TESMs had no effect. Whole-mount immunofluorescence corroborated these findings and showed TTN-positive and cross-striated myofibers exclusively in control and in *micro-dystrophin*-treated *DMD* knock down 3D-TESMs, while none was present in the *DMD* knock downs and the knock down in the presence of *GFP* (Fig. 4D–F). Interestingly, when analyzing the 3D-TESMs with *DMD* knock downs in the presence of *GFP*, we observed elongated multinucleated GFP-positive cells that resembled short myofibers that lacked striation, suggesting that *DMD* knock down allowed myogenic differentiation but resulted in a lack of maturation or a loss of matured myofibers (Fig. 4D).

Taken together, these results confirm the specificity of the knock down approach for the modelling of muscular dystrophies in 3D-TESMs, and they demonstrate the utility of the shRNA 3D-TESM system to predict the therapeutic value of novel treatment options in human muscle in vitro.

## Discussion

Tissue-engineered 3D-disease models are becoming more prevalent as these can better mimic tissue complexity and thereby become more representative for human disease as compared to 2D models or heterologous cells. The use of such 3D cell culture models is often incompatible with transfection or transduction protocols due to low targeting efficiencies [12, 30]. Utilizing the hydrogel as a delivery vehicle for lentiviral particles, we obtained an increased targeting efficiency that allowed efficient transduction of cells within the 3D-TESM tissues.

The use of both fibrin or collagen-based matrixes for cellular delivery of DNA constructs has been explored previously both in vitro and in vivo [31, 32]. Fibrin matrixes have been shown capable of enveloping viral particles, resulting in increased stability and prolonged life span of the viral particles while encapsulated within the polymerized matrix [31, 33]. The use of collagen-based matrixes is commonly described for the transfer of naked DNA such as plasmids or DNA minicircles that are able to interact through their negatively charged backbone [30, 34]. Utilization of hydrogel-based delivery of DNA could therefore represent a viable method to enable higher-throughput experiments in 3D-cell culture systems and also may extend to other difficult-to-target 3D models that utilize collagen or fibrin matrixes as a structural basis such as engineered heart tissues, droplet cultures, or other organ-on-chip platforms.

Combining lentiviral shRNAs with a hydrogel-based delivery method, we aimed to develop a platform that is capable of recapitulating muscular dystrophies in 3D-TESMs. Inducing genetic deficiency of *DMD* or *CAPN3* as a proof of principle, a significantly decreased contractile force of the 3D-TESMs was observed shortly after inducing myogenesis. Using mass spectrometry to obtain proteomic profiles, distinct proteomic signatures were obtained, corresponding with previous studies performed with patient biopsies or animal models of Duchenne muscular dystrophy and LGMD2A. Calpain-3 is a calcium-dependent protease responsible for the cleavage of cytoskeletal proteins and actin filaments in skeletal muscle; it is therefore a prerequisite for the maintenance and remodeling of healthy skeletal muscle [23, 35, 36]. While the exact molecular mechanisms underlying the pathology of LGMD2A remain unclear, CAPN3-deficient muscles have been found to exhibit structural abnormalities of the sarcomere and cytoskeleton [22, 23, 37, 38]. The 3D-TESMs recapitulate these abnormalities, as we observed a dysregulation of several ankyrin proteins (ANK2 and ANK3), ankyrin repeat proteins (ANKRD1, ANKRD2, and ANKS1A), and catenin (CTNNA1, CTNNB1, and CTNND1) isoforms which provide structural integrity to the cytoskeleton and are known substrates of Calpain-3. In addition, we observed a downregulation of multiple PSMA and PSMB isoforms, which compose the active site of the 20S proteasome, resembling previous findings made in Calpain-3-deficient (C3KO) mice and immortalized human CAPN3-deficient cell lines by [36].

Like previous studies that used DMD-deficient primary myoblasts or CRISPR/Cas9-induced *DMD* knockouts to model Duchenne muscular dystrophy in 3D-TESMs, the knock down of *DMD* resulted in a significantly reduced force-generating capacity [39–41]. Concurrent with this reduction in contractile properties, we found a significant reduction of the expression of proteins involved in the structure and function of the sarcomere (MYHs, TTN, NEB, OBSCN, ATP1A2, ATP2A1), as well as of SGCA, SGCD, and SGCG, which are part of the sarcoglycan complex that directly connects dystrophin to the surrounding ECM [24, 42]. In addition, we observed an upregulation of the expression of several annexin isoforms (ANXA1-5) and tubulins (TUBB2A and TUBB2B), which are commonly associated with membrane repair, a pathway that is upregulated in muscle tissues from Duchenne patients and the *mdx* mouse model [24, 43]. Another key aspect related to the pathogenicity of Duchenne muscular dystrophy is the progressive conversion of skeletal muscle tissue into fibrotic tissue [24, 44]. Immune cell infiltration is thought to play a role in this process. Although the 3D model employed here lacks immune cells, we nevertheless found an increased expression of COL4A1, COL4A2, COL6A1, LAMA1, and LAMA2 and other ECM proteins that are commonly associated with a fibrotic phenotype, suggesting the existence of an immune cell-independent pathway underlying fibrosis in Duchenne muscular dystrophy [24, 44].

Inducing the expression of *micro-dystrophin* in conjunction with the *DMD-*targeting shRNA resulted in partial restoration of contractile force. Previous characterizations of *micro-dystrophin* have predominantly been performed in vivo using mouse or canine models [45, 46]. Similar to the results obtained in the present study with the 3D-TESM model, these studies have shown an improvement in skeletal muscle architecture and a partial restoration of muscle tissue performance upon systemic expression of *micro-dystrophin* [46–48]. Ongoing phase I/II clinical trials corroborate these results, observing an overall reduction of creatine kinase (CK) levels and a modest improvement of muscle function (NCAA score) in four young patients with Duchenne muscular dystrophy [17]. The similarity between the extent of functional rescue of muscle performance between 3D-TESMs, animal models, and human patients highlights the predictive value of the shRNA-mediated *DMD* knock down 3D-TESM model for testing novel therapeutic options.

The knock down of *MSTN* did not result in any significant differences in contractile force, tissue quality, or proteomic changes compared to control 3D-TESMs. *MSTN* is known to affect muscle hypertrophy and is not required during the initial stages of myogenesis [49, 50]. It is therefore possible that extending the culture period might result in an increased force-generating capacity, which should be tested in future studies. Such a result is suggested by the increased twitch and tetanic force, although not significant, in *MSTN* knock down 3D-TESMs at the latest timepoint (Fig. 2).

3D-organ-on-a-chip technologies are expected to provide a crucial stepping stone for the development of novel therapies and provide an avenue to reduce animal testing [10]. For neuromuscular disorders in particular, the possibility to measure the contractile force of human cells in vitro allows for the functional evaluation of therapeutic interventions [8, 9]. It has become increasingly clear that isogenic disease models are important to correct for genetic background and to detect disease-related pathology rather than random variability [11]. To generate such disease models, elaborate procedures are required involving gene editing of primary cells, possibly via hiPSCs. The approach described here provides a rapid method to generate isogenic disease models from hiPSCs that can be applied to any existing hiPSC line available. We anticipate that it can also be applied to 3D-TESMs generated from primary human myoblasts, as we recently found that these have similar properties as the hiPSC-derived ones [51].

### Limitations and future directions

The protocol outlined here has a number of limitations. The knock down approach can only be used to model reduction of gene products, not their total absence or gain of functions. The knock down is present before the start of differentiation. To evaluate possible effects of knock down on myotube formation, the fusion index should be calculated, which is the most accurate in a 2D differentiation, because of overlapping planes when analyzing 3D tissues. Whether a knock down will affect myotube formation or not, the fact that the gene deficiency is induced prior to myogenic differentiation mimics the situation in patients in which the gene defect is inherited.

Future developments should be focused on improving the skeletal muscle on a chip model, such as (1) inclusion of additional cell types that are also present in native skeletal muscle tissue, (2) improvement of throughput to allow medium-sized screens (for a first step towards this goal, see Iuliano et al. [52]), and (3) longer-term culture and fiber maturation to assess longer-term recapitulation of phenotypes and the ability to reverse these using novel treatment options [8, 9, 26, 51–53]. Such improvements should be standardized, and the models should be validated in order to use these for the preclinical evaluation of efficacy and safety of treatment options. As the organ-on-a-chip field is progressing rapidly, we anticipate that much progress on these points will be made in the near future. The value of organ-on-a-chip models in predicting clinical phenotypes and responses to treatments is being increasingly appreciated, for example, as indicated by the recent acceptance by the FDA of using data obtained with organ on a chip models rather than with animal models for drug development [54].

### Supplementary Information

**Additional file 1: Figure S1.** Effect of lentiviral transduction on fusion index of Myogenic Progenitor Cells in 2D. **Figure S2.** Validation of shRNA-mediated knock down of *CAPN3* in 3D-tissue-engineered-skeletal-muscle. **Figure S3.** Validation of shRNA-mediated knock down of *DMD* in 3D-tissue-engineered-skeletal-muscle. **Figure S4.** Validation of shRNA-mediated knock down of *MSTN* in 3D-tissue-engineered-skeletal-muscle. **Figure S5.** Effect of shRNA-mediated knock down on fusion index of Myogenic Progenitor Cells. **Figure S6.** Diameter and alignment of myofibers in the 3D-tissue-engineered-skeletal-muscles analyzed in Figs. 2 and 3. **Figure S7.** Bright field microscopy of 3D-tissue-engineered-skeletal-muscles in culture. **Figure S8.** Proteome assessment of myogenesis in 3D-tissue-engineered-skeletal-muscle. **Figure S9.** Quality control of the proteomic analysis of 3D-tissue-engineered-skeletal-muscles. **Table S1.** hiPSC lines used in this study. **Table S2.** Primer list. **Supplemental methods.** Proteomic analysis. Statistics.

## Data Availability

The mass spectrometry proteomics data have been deposited to the ProteomeXchange Consortium via the PRIDE partner repository with the dataset identifier PXD042227. This dataset will be available upon publication.

## Abbreviations

3D-TESM: 3D-tissue-engineered-skeletal muscle
hiPSC: Human-induced pluripotent stem cell
MPC: Myogenic progenitor cell

## References

[1] Cohen E, Bonne G, Rivier F, Hamroun D (2021). The 2022 version of the gene table of neuromuscular disorders (nuclear genome). Neuromuscul Disord.

[2] Jablonka S, Hennlein L, Sendtner M (2022). Therapy development for spinal muscular atrophy: perspectives for muscular dystrophies and neurodegenerative disorders. Neurol Res Pract.

[3] Motohashi N, Shimizu-Motohashi Y, Roberts TC, Aoki Y (2019). Potential therapies using myogenic stem cells combined with bio-engineering approaches for treatment of muscular dystrophies. Cells.

[4] Vita G, Vita GL, Musumeci O, Rodolico C, Messina S (2019). Genetic neuromuscular disorders: living the era of a therapeutic revolution. Part 2: diseases of motor neuron and skeletal muscle. Neurol Sci.

[5] Jalal S, Dastidar S, Tedesco FS (2021). Advanced models of human skeletal muscle differentiation, development and disease: three-dimensional cultures, organoids and beyond. Curr Opin Cell Biol.

[6] Zschüntzsch J, Meyer S, Shahriyari M, Kummer K, Schmidt M, Kummer S (2022). The evolution of complex muscle cell in vitro models to study pathomechanisms and drug development of neuromuscular disease. Cells.

[7] Speciale AA, Ellerington R, Goedert T, Rinaldi C (2020). Modelling neuromuscular diseases in the age of precision medicine. J Pers Med.

[8] Khodabukus A, Prabhu N, Wang J, Bursac N. In vitro tissue-engineered skeletal muscle models for studying muscle physiology and disease. Adv Healthc Mater. 2018. Available from: https://onlinelibrary.wiley.com/doi/full/10.1002/adhm.201701498. 10.1002/adhm.201701498 PMC6105407 29696831

[9] Moyle LA, Jacques E, Gilbert PM (2020). Engineering the next generation of human skeletal muscle models: from cellular complexity to disease modeling. Curr Opin Biomed Eng.

[10] Van Den Berg A, Mummery CL, Passier R, Van der Meer AD (2019). Personalised organs-on-chips: functional testing for precision medicine. Lab Chip.

[11] Volpato V, Webber C (2020). Addressing variability in iPSC-derived models of human disease: guidelines to promote reproducibility. Dis Model Mech.

[12] Menche C, Farin HF (2021). Strategies for genetic manipulation of adult stem cell-derived organoids. Exp Mol Med.

[13] van der Wal E, Herrero-Hernandez P, Wan R, Broeders M, in ‘t Groen SLM, van Gestel TJM (2018). Large-scale expansion of human iPSC-derived skeletal muscle cells for disease modeling and cell-based therapeutic strategies. Stem Cell Rep.

[14] van der Wal E, Bergsma AJ, van Gestel TJM, in ‘t Groen SLM, Zaehres H, Araúzo-Bravo MJ (2017). GAA deficiency in Pompe disease is alleviated by exon inclusion in iPSC-derived skeletal muscle cells. Mol Ther Nucleic Acids.

[15] Iuliano A, van der Wal E, Ruijmbeek CWB, in ‘t Groen SLM, Pijnappel WWMP, de Greef JC (2020). Coupling 3D printing and novel replica molding for in house fabrication of skeletal muscle tissue engineering devices. Adv Mater Technol.

[16] Kimura E, Han JJ, Li S, Fall B, Ra J, Haraguchi M (2008). Cell-lineage regulated myogenesis for dystrophin replacement: a novel therapeutic approach for treatment of muscular dystrophy. Hum Mol Genet.

[17] Mendell JR, Sahenk Z, Lehman K, Nease C, Lowes LP, Miller NF (2020). Assessment of systemic delivery of rAAVrh74.MHCK7.micro-dystrophin in children with duchenne muscular dystrophy: a nonrandomized controlled trial. JAMA Neurol.

[18] Yang X, Boehm JS, Yang X, Salehi-Ashtiani K, Hao T, Shen Y (2011). A public genome-scale lentiviral expression library of human ORFs. Nat Methods.

[19] Liang Q, Catalano F, Vlaar EC, Pijnenburg JM, Stok M, van Helsdingen Y (2022). IGF2-tagging of GAA promotes full correction of murine Pompe disease at a clinically relevant dosage of lentiviral gene therapy. Mol Ther Methods Clin Dev.

[20] Demichev V, Messner CB, Vernardis SI, Lilley KS, Ralser M (2019). DIA-NN: neural networks and interference correction enable deep proteome coverage in high throughput. Nat Methods.

[21] Nolte H, MacVicar TD, Tellkamp F, Krüger M (2018). Instant clue: a software suite for interactive data visualization and analysis. Sci Rep.

[22] Kramerova I, Kudryashova E, Venkatraman G, Spencer MJ (2005). Calpain 3 participates in sarcomere remodeling by acting upstream of the ubiquitin–proteasome pathway. Hum Mol Genet.

[23] Lasa-Elgarresta J, Mosqueira-Martín L, Naldaiz-Gastesi N, Sáenz A, de Munain AL, Vallejo-Illarramendi A (2019). Calcium mechanisms in limb-girdle muscular dystrophy with CAPN3 mutations. Int J Mol Sci.

[24] Gargan S, Dowling P, Zweyer M, Henry M, Meleady P, Swandulla D (2022). Proteomic identification of markers of membrane repair, regeneration and fibrosis in the aged and dystrophic diaphragm. Life.

[25] Schiaffino S, Dyar KA, Ciciliot S, Blaauw B, Sandri M (2013). Mechanisms regulating skeletal muscle growth and atrophy. FEBS J.

[26] Khodabukus A (2021). Tissue-engineered skeletal muscle models to study muscle function, plasticity, and disease. Front Physiol.

[27] Selvaraj S, Mondragon-Gonzalez R, Xu B, Magli A, Kim H, Lainé J (2019). Screening identifies small molecules that enhance the maturation of human pluripotent stem cell-derived myotubes. Elife.

[28] Vandenburgh H, Shansky J, Benesch-Lee F, Barbata V, Reid J, Thorrez L (2008). Drug-screening platform based on the contractility of tissue-engineered muscle. Muscle Nerve.

[29] Wang L, Shansky J, Vandenburgh H (2013). Induced formation and maturation of acetylcholine receptor clusters in a defined 3D bio-artificial muscle. Mol Neurobiol.

[30] Di Cesare PE, Frenkel SR, Carlson CS, Fang C, Liu C (2006). Regional gene therapy for full-thickness articular cartilage lesions using naked DNA with a collagen matrix. J Orthop Res.

[31] Kidd ME, Shin S, Shea LD (2012). Fibrin hydrogels for lentiviral gene delivery in vitro and in vivo. J Control Release.

[32] Raut SD, Lei P, Padmashali RM, Andreadis ST. Fibrin-mediated lentivirus gene transfer: implications for lentivirus microarrays. J Control Release. 2010;144:213. [cited 2024 Feb 5]. Available from: https://www.ncbi.nlm.nih.gov/pmc/articles/PMC2868957/. 10.1016/j.jconrel.2010.02.009 PMC2868957 20153386

[33] Padmashali RM, Andreadis ST (2011). Engineering fibrinogen-binding VSV-G envelope for spatially- and cell-controlled lentivirus delivery through fibrin hydrogels. Biomaterials.

[34] Scherer F, Schillinger U, Putz U, Stemberger A, Plank C (2002). Nonviral vector loaded collagen sponges for sustained gene delivery in vitro and in vivo. J Gene Med.

[35] Hata S, Doi N, Shinkai-Ouchi F, Ono Y (2020). A muscle-specific calpain, CAPN3, forms a homotrimer. Biochim Biophys Acta Proteins Proteom.

[36] Lasa-Elgarresta J, Mosqueira-Martín L, González-Imaz K, Marco-Moreno P, Gerenu G, Mamchaoui K (2022). Targeting the ubiquitin-proteasome system in limb-girdle muscular dystrophy with CAPN3 mutations. Front Cell Dev Biol.

[37] Kramerova I, Kudryashova E, Wu B, Ottenheijm C, Granzier H, Spencer MJ (2008). Novel role of calpain-3 in the triad-associated protein complex regulating calcium release in skeletal muscle. Hum Mol Genet.

[38] Sáenz A, Azpitarte M, Armañanzas R, Leturcq F, Alzualde A, Inza I (2008). Gene expression profiling in limb-girdle muscular dystrophy 2A. PLoS One.

[39] Ebrahimi M, Lad H, Fusto A, Tiper Y, Datye A, Nguyen CT (2021). De novo revertant fiber formation and therapy testing in a 3D culture model of Duchenne muscular dystrophy skeletal muscle. Acta Biomater.

[40] Maffioletti SM, Sarcar S, Henderson ABH, Mannhardt I, Pinton L, Moyle LA (2018). Three-dimensional human iPSC-derived artificial skeletal muscles model muscular dystrophies and enable multilineage tissue engineering. Cell Rep.

[41] Smith ASST, Luttrell SM, Dupont J-BB, Gray K, Lih D, Fleming JW (2022). High-throughput, real-time monitoring of engineered skeletal muscle function using magnetic sensing. J Tissue Eng.

[42] Young CS, Hicks MR, Ermolova NV, Nakano H, Jan M, Younesi S (2016). A single CRISPR-Cas9 deletion strategy that targets the majority of DMD patients restores dystrophin function in hiPSC-derived muscle cells. Cell Stem Cell.

[43] Croissant C, Carmeille R, Brévart C, Bouter A (2021). Annexins and membrane repair dysfunctions in muscular dystrophies. Int J Mol Sci.

[44] Klingler W, Jurkat-Rott K, Lehmann-Horn F, Schleip R (2012). The role of fibrosis in Duchenne muscular dystrophy. Acta Myol.

[45] Duan D (2018). Systemic AAV micro-dystrophin gene therapy for Duchenne muscular dystrophy. Mol Ther.

[46] Shin JH, Pan X, Hakim CH, Yang HT, Yue Y, Zhang K (2013). Microdystrophin ameliorates muscular dystrophy in the canine model of duchenne muscular dystrophy. Mol Ther.

[47] Hamm SE, Fathalikhani DD, Bukovec KE, Addington AK, Zhang H, Perry JB (2021). Voluntary wheel running complements microdystrophin gene therapy to improve muscle function in mdx mice. Mol Ther Methods Clin Dev.

[48] Potter RA, Griffin DA, Heller KN, Peterson EL, Clark EK, Mendell JR (2021). Dose-escalation study of systemically delivered rAAVrh74.MHCK7.micro-dystrophin in the mdx mouse model of Duchenne muscular dystrophy. Hum Gene Ther.

[49] Matsakas A, Otto A, Elashry MI, Brown SC, Patel K (2010). Altered primary and secondary myogenesis in the myostatin-null mouse. Rejuvenation Res.

[50] Sharma M, McFarlane C, Kambadur R, Kukreti H, Bonala S, Srinivasan S (2015). Myostatin: expanding horizons. IUBMB Life.

[51] Van Der Wal E, Iuliano A, In ‘t Groen SLM, Bholasing AP, Priesmann D, Sharma P (2023). Stem cell reports resource highly contractile 3D tissue engineered skeletal muscles from human iPSCs reveal similarities with primary myoblast-derived tissues. Stem Cell Rep.

[52] Iuliano A, Haalstra M, Raghuraman R, Bielawski K, Bholasing AP, van der Wal E, et al. Real-time and multichannel measurement of contractility of hiPSC-derived 3D skeletal muscle using fiber optics-based sensing. Adv Mater Technol. 2023:2300845. Available from: https://onlinelibrary.wiley.com/doi/full/10.1002/admt.202300845. Cited 2023 Oct 23.

[53] Moyle LA, Davoudi S, Gilbert PM (2022). Innovation in culture systems to study muscle complexity. Exp Cell Res.

[54] Wadman M (2023). FDA no longer has to require animal testing for new drugs. Science.

