# Supplementary material for: A knock down strategy for rapid, generic, and versatile modelling of muscular dystrophies in 3D-tissue-engineered-skeletal muscle
Source: Skelet Muscle. 2024 Feb 22;14:3. doi: 10.1186/s13395-024-00335-5 (PMC10882755; doi:10.1186/s13395-024-00335-5)
Supplement: Supplementary file 1 — Additional file 1: Figure S1. Effect of lentiviral transduction on fusion index of Myogenic Progenitor Cells in 2D. Figure S2. Validation of shRNA-mediated knock down of CAPN3 in 3D-tissue-engineered-skeletal-muscle. Figure S3. Validation of shRNA-mediated knock down of DMD in 3D-tissue-engineered-skeletal-muscle. Figure S4. Validation of shRNA-mediated knock down of MSTN in 3D-tissue-engineered-skeletal-muscle. Figure S5. Effect of shRNA-mediated knock down on fusion index of Myogenic Progenitor Cells. Figure S6. Diameter and alignment of myofibers in the 3D-tissue-engineered-skeletal-muscles analyzed in Figs. 2 and 3. Figure S7. Bright field microscopy of 3D-tissue-engineered-skeletal-muscles in culture. Figure S8. Proteome assessment of myogenesis in 3D-tissue-engineered-skeletal-muscle. Figure S9. Quality control of the proteomic analysis of 3D-tissue-engineered-skeletal-muscles. Table S1. hiPSC lines used in this study. Table S2. Primer list. Supplemental methods. Proteomic analysis. Statistics. [file 13395_2024_335_MOESM1_ESM.pdf]

A

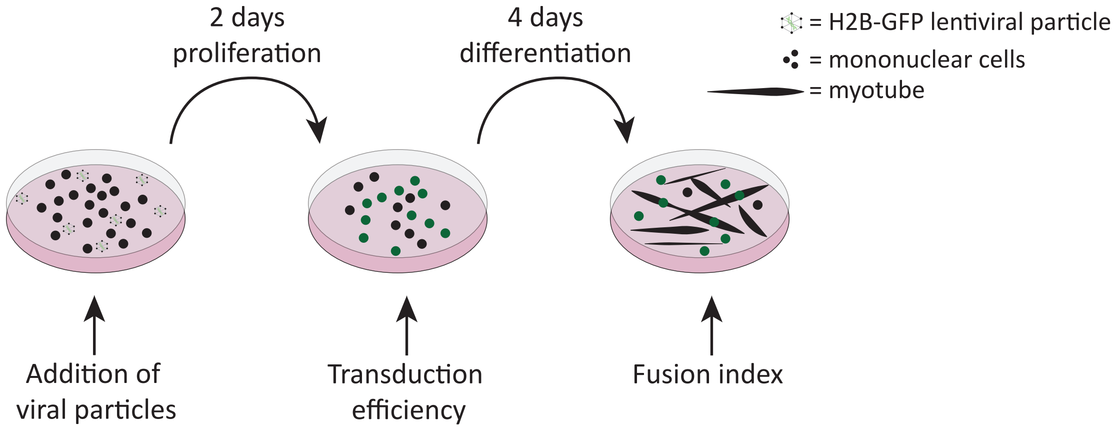

B

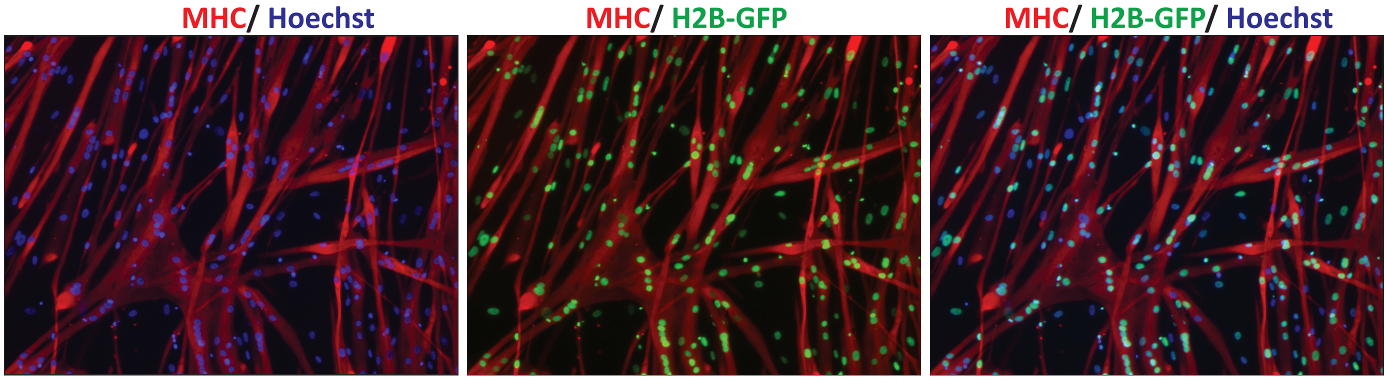

C

	Transduced MPCs	untransduced MPCs
Targeting efficiency	88% ±4%	0% ±0%
Fusion index	79% ±6%	84% ±2%

Figure S1 Effect of lentiviral transduction on fusion index of Myogenic Progenitor Cells in 2D.

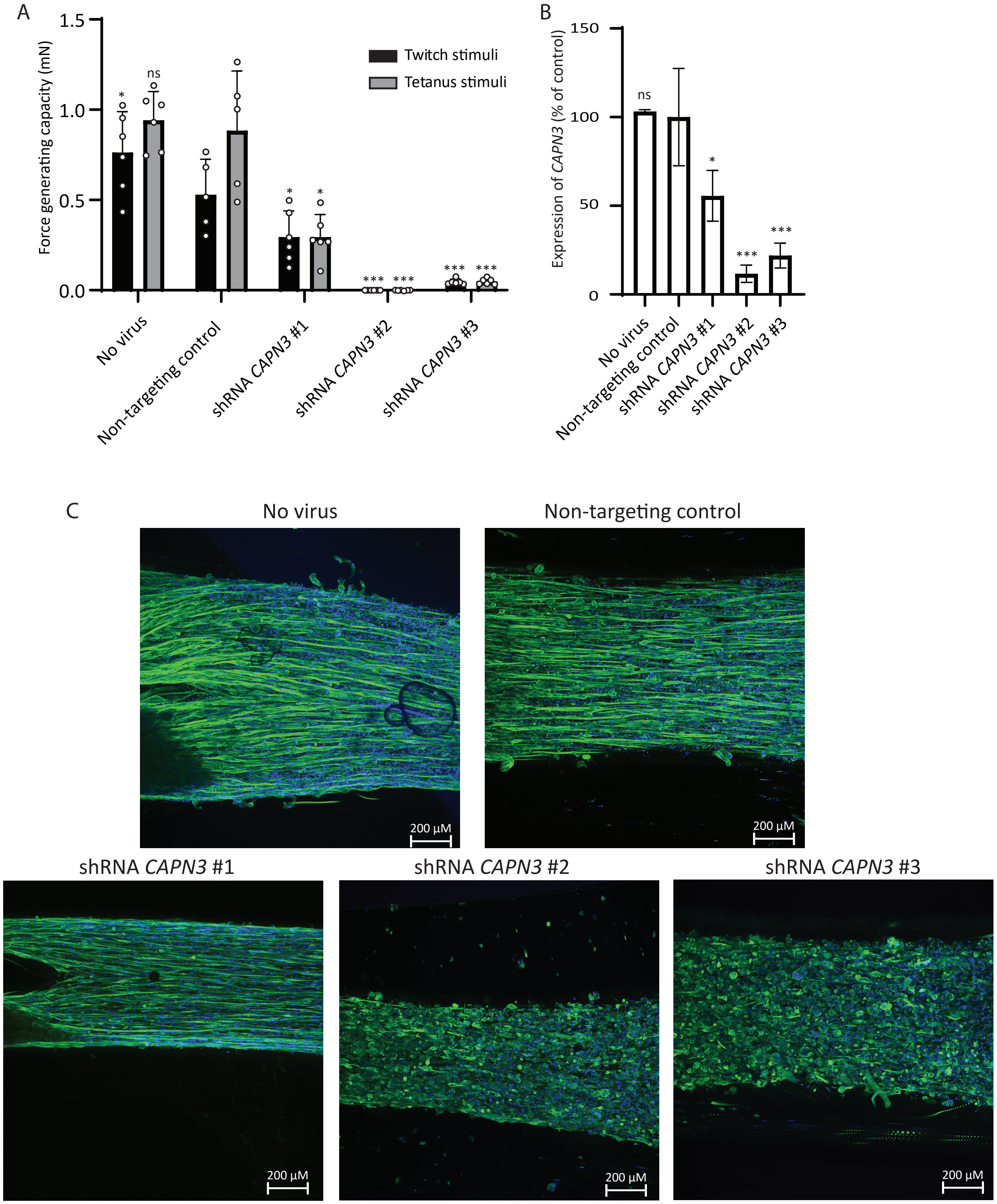

Figure S2 Validation of shRNA-mediated knock down of *CAPN3* in 3D-tissue-engineered-skeletal-muscle

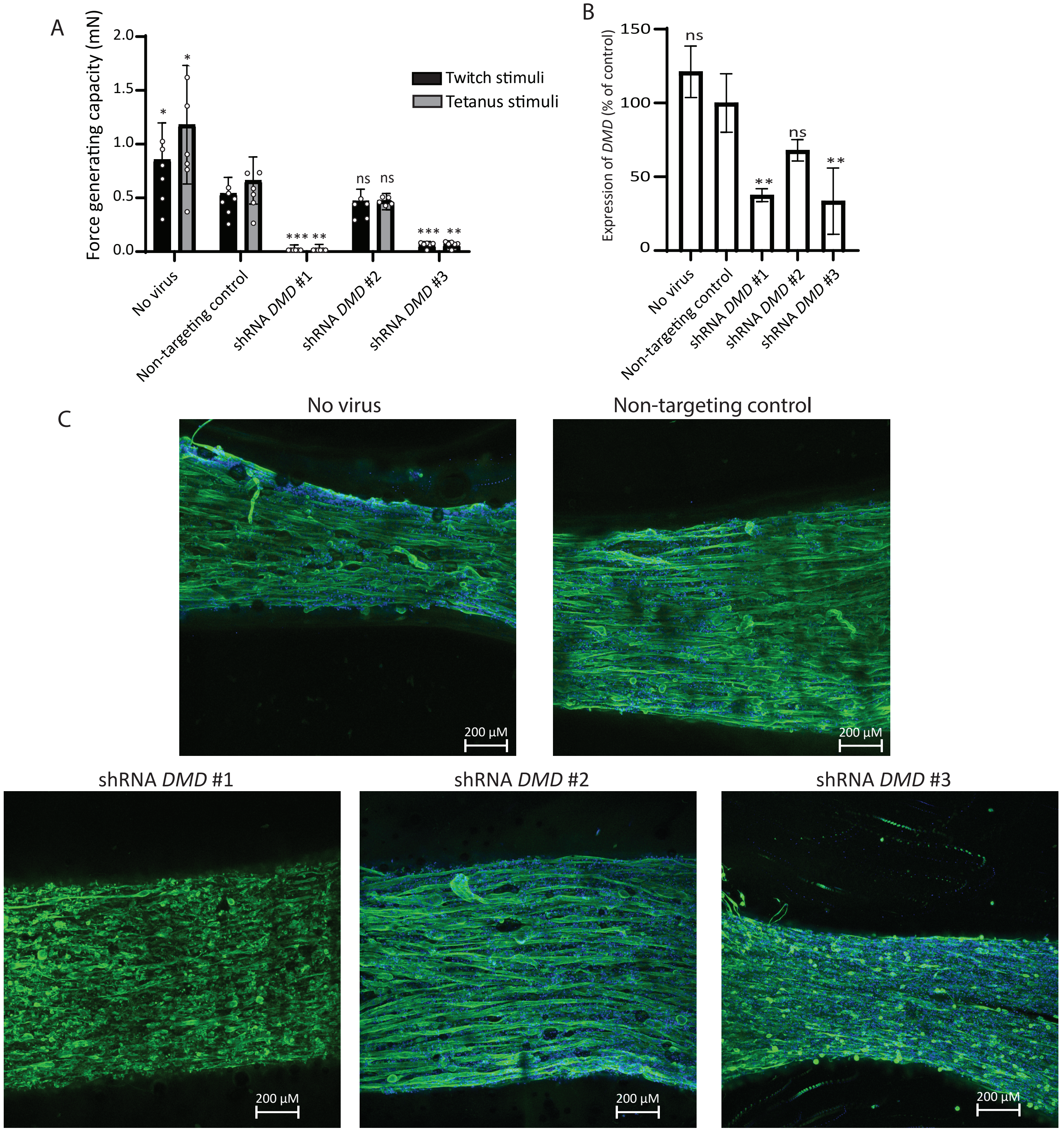

Figure S3 Validation of shRNA-mediated knock down of *DMD* in 3D-tissue-engineered-skeletal-muscle

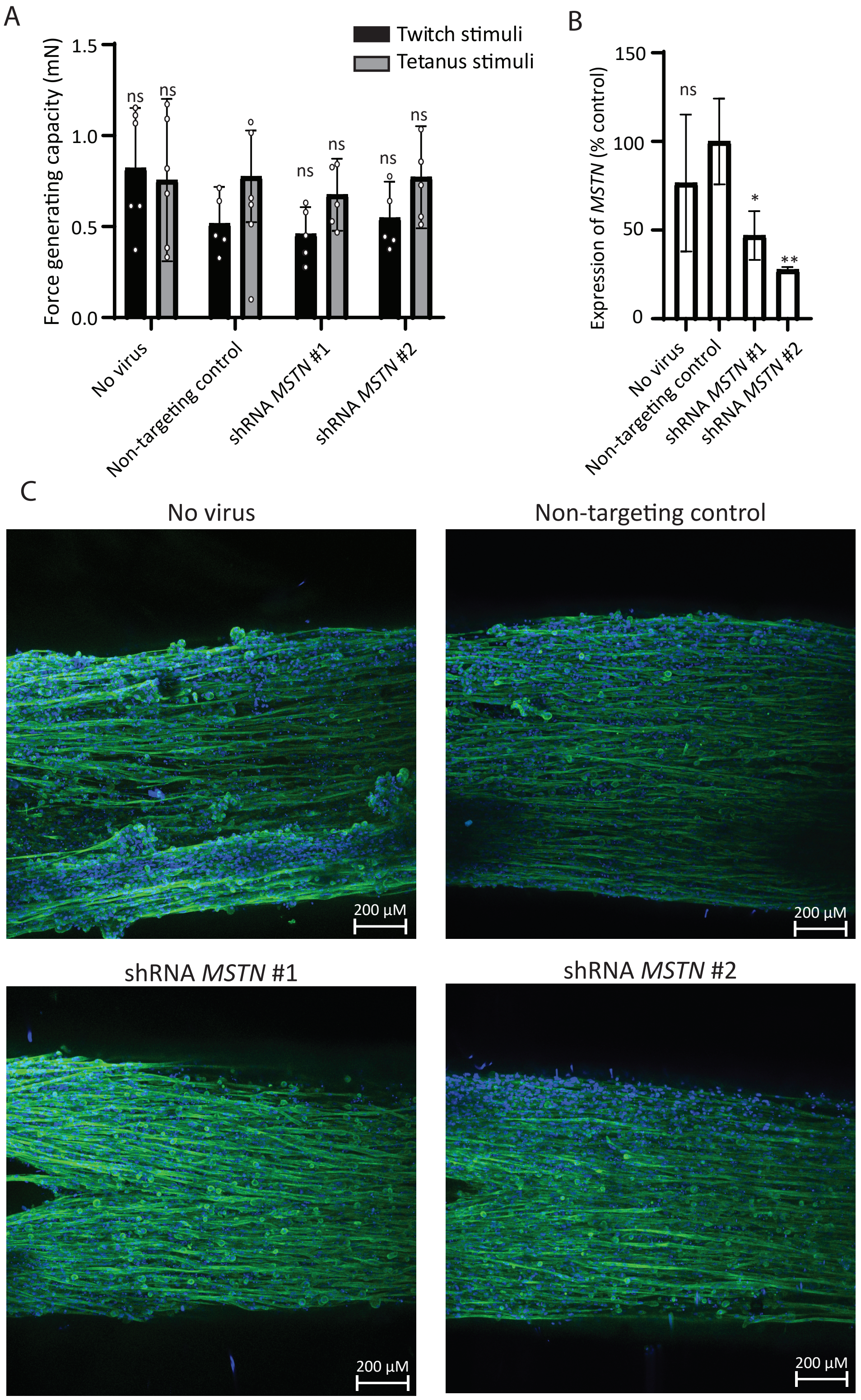

Figure S4 Validation of shRNA-mediated knock down of *MSTN* in 3D-tissue-engineered-skeletal-muscle

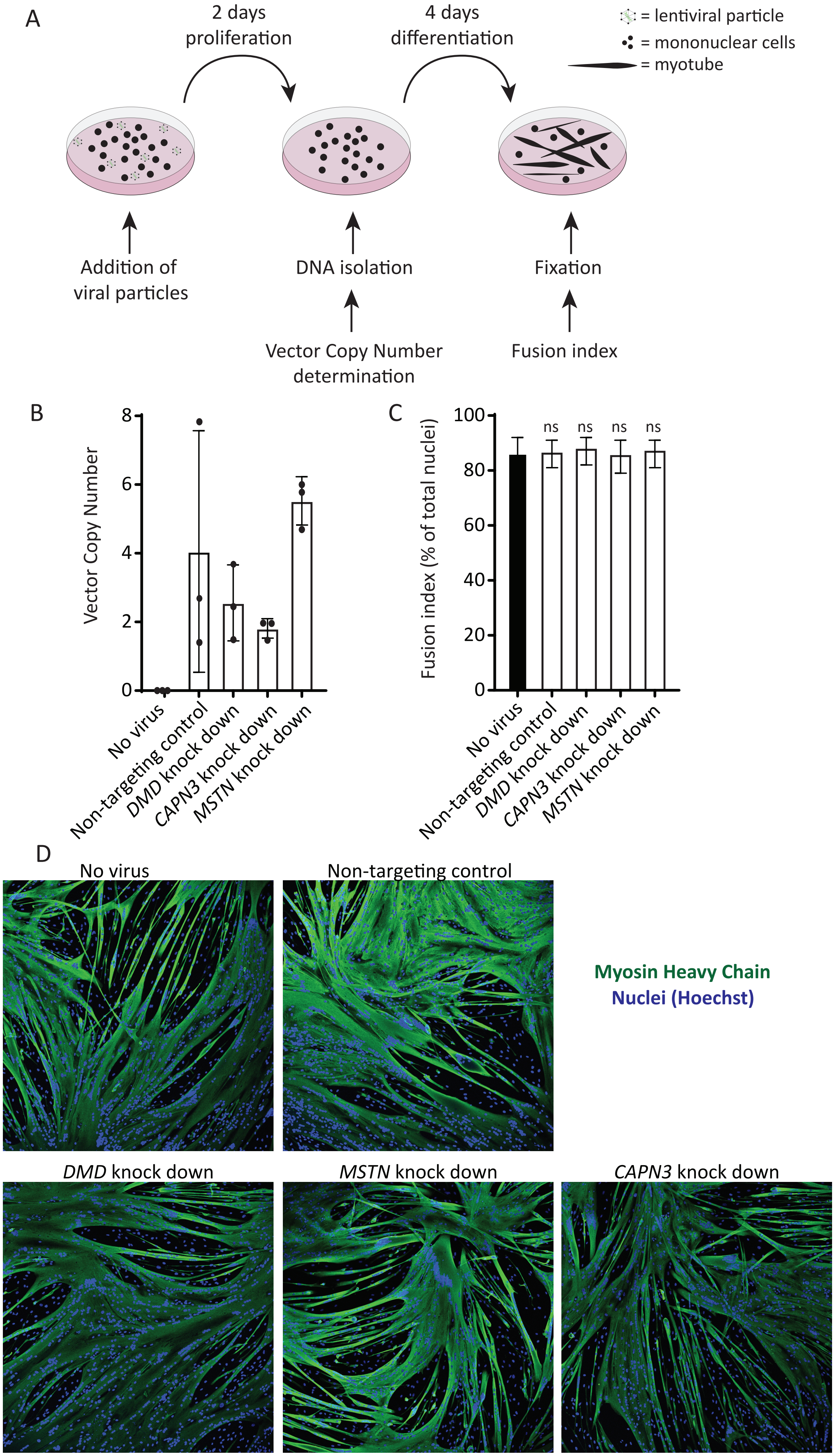

Figure S5 Effect of shRNA-mediated knock down on fusion index of Myogenic Progenitor Cells.

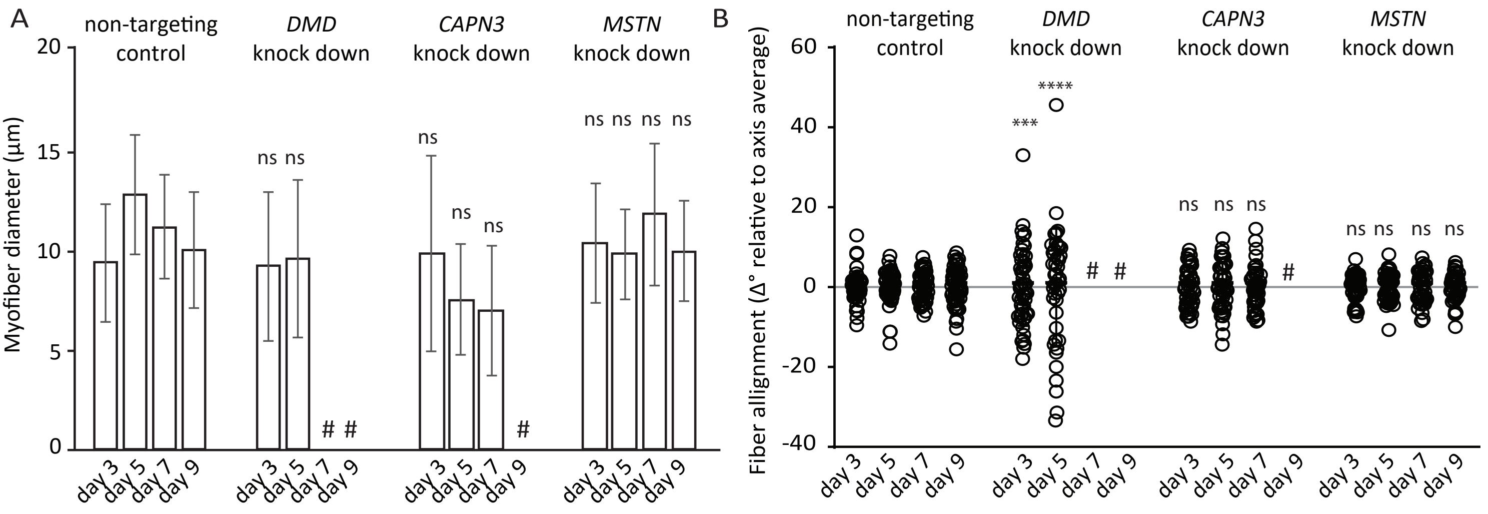

Figure S6 Diameter and alignment of myofibers in the 3D-tissue-engineered-skeletal-muscles analyzed in Figures 2 and 3.

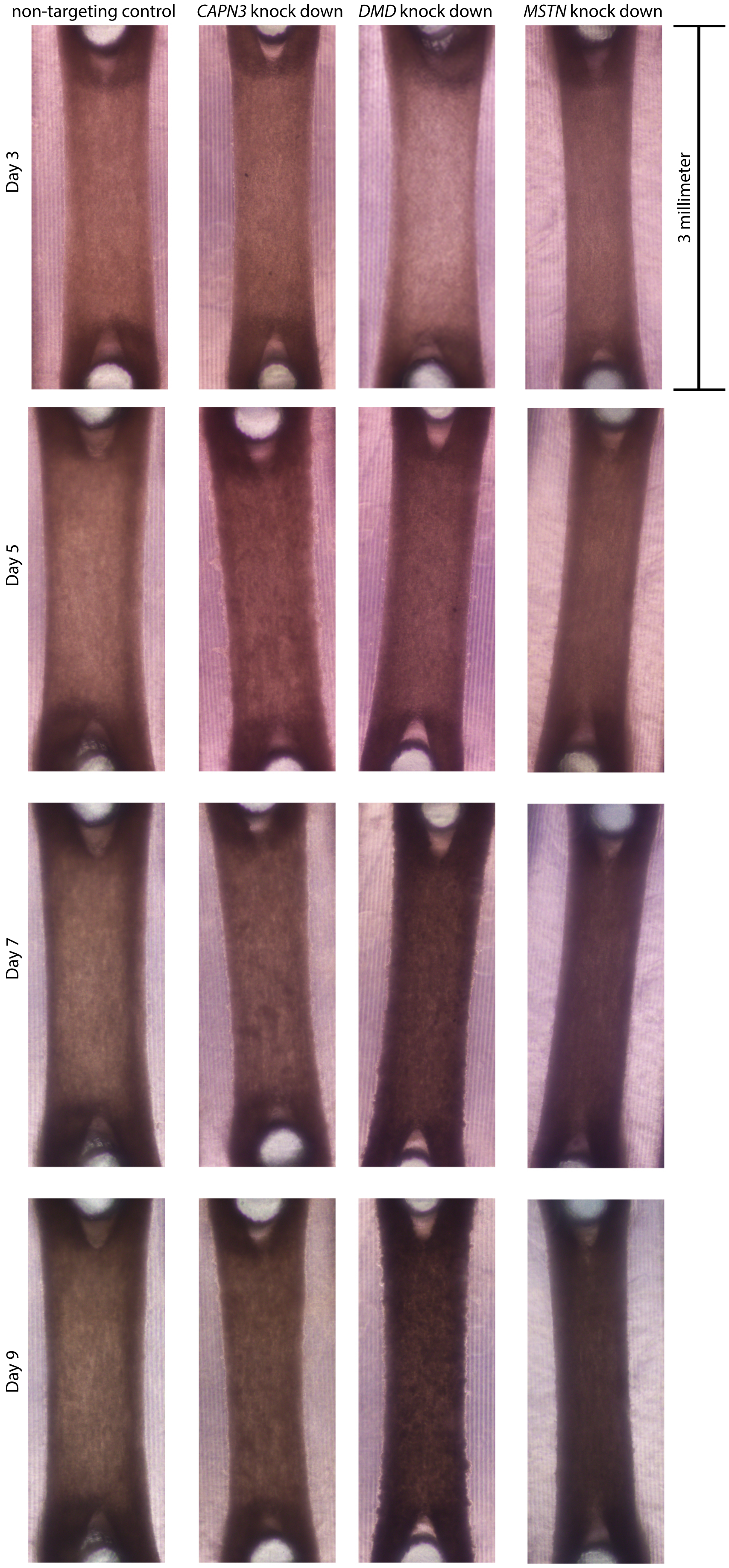

Figure S7 Bright field microscopy of 3D-tissue-engineered-skeletal-muscles in culture.

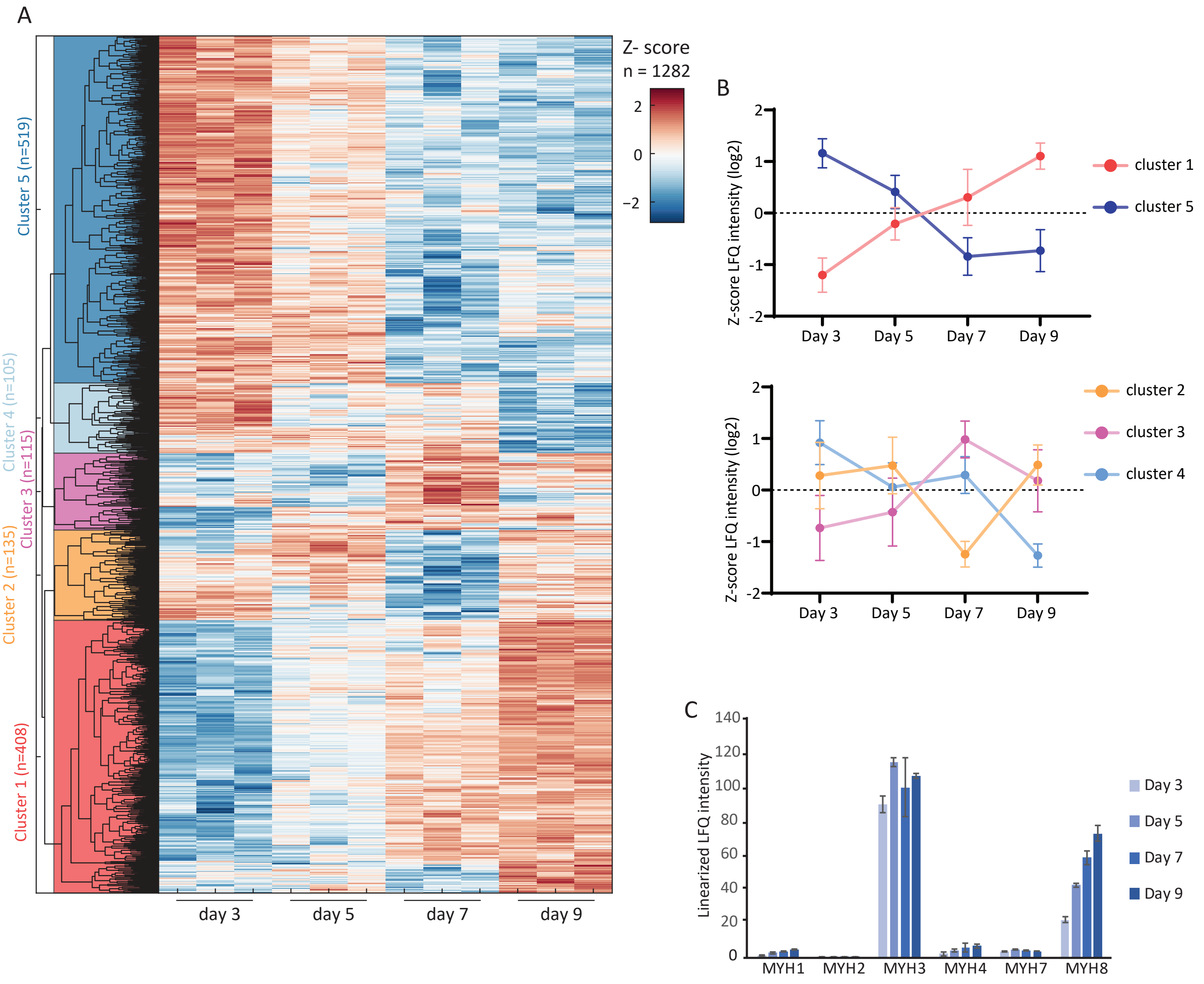

D

Top 5 upregulated pathways (Cluster 1)

GO: term ID	GO: term description	strength	FDR
GO:0015980	Energy derivation by oxidation of organic compounds	1.13	8.02E-39
GO:0045333	Cellular respiration	1.21	1.86E-36
GO:0006936	Muscle contraction	0.89	8.67E-18
GO:0006099	Tricarboxylic acid cycle	1.44	6.54E-16
GO:0006006	Glucose metabolic process	1.01	4.78E-13

E

Top 5 downregulated pathways (Cluster 5)

GO: term ID	GO: term description	strength	FDR
GO:0043488	Regulation of mrna stability	0.75	3.42E-09
GO:0034250	Positive regulation of cellular amide metabolic process	0.75	3.84E-08
GO:0006401	RNA catabolic process	0.65	4.67E-08
GO:0050658	rRNA processing	0.67	0.000000188
GO:1903312	Negative regulation of mrna metabolic process	0.84	0.00000273

F

	day 3	day 5	day 7	day 9
IGF2R	100%	81%	57%	64%
MYOF	100%	78%	63%	60%
MYORG	100%	55%	72%	47%
MYOG	96%	100%	67%	39%
MUSTN1	60%	80%	100%	53%
TMOD1	78%	90%	100%	97%
TMOD3	100%	85%	76%	46%
ACTN3	30%	57%	79%	100%
ACTN4	100%	71%	58%	52%
MYH1	27%	55%	73%	100%
MYH3	78%	100%	86%	93%
MYH7	76%	100%	93%	82%
MYH8	30%	58%	81%	100%
COL1A1	69%	64%	56%	100%
COL4A2	70%	82%	76%	100%
COL5A1	17%	33%	54%	100%
COL6A1	100%	73%	63%	69%
COL15A1	34%	53%	72%	100%
MB	4%	14%	44%	100%
CKM	34%	55%	72%	100%
DMD	39%	56%	79%	100%
OBSCN	53%	74%	85%	100%
NEB	68%	86%	100%	93%

Color scale: 100%, 95%, 90%, 85%, 80%, 75%, 70%, 65%, 60%, 55%, 50%, 45%, 40%, 35%, 30%, 25%, 20%, 15%, 10%, 5%, 0%

Figure S8 Proteome assessment of myogenesis in 3D-tissue-engineered-skeletal-muscle.

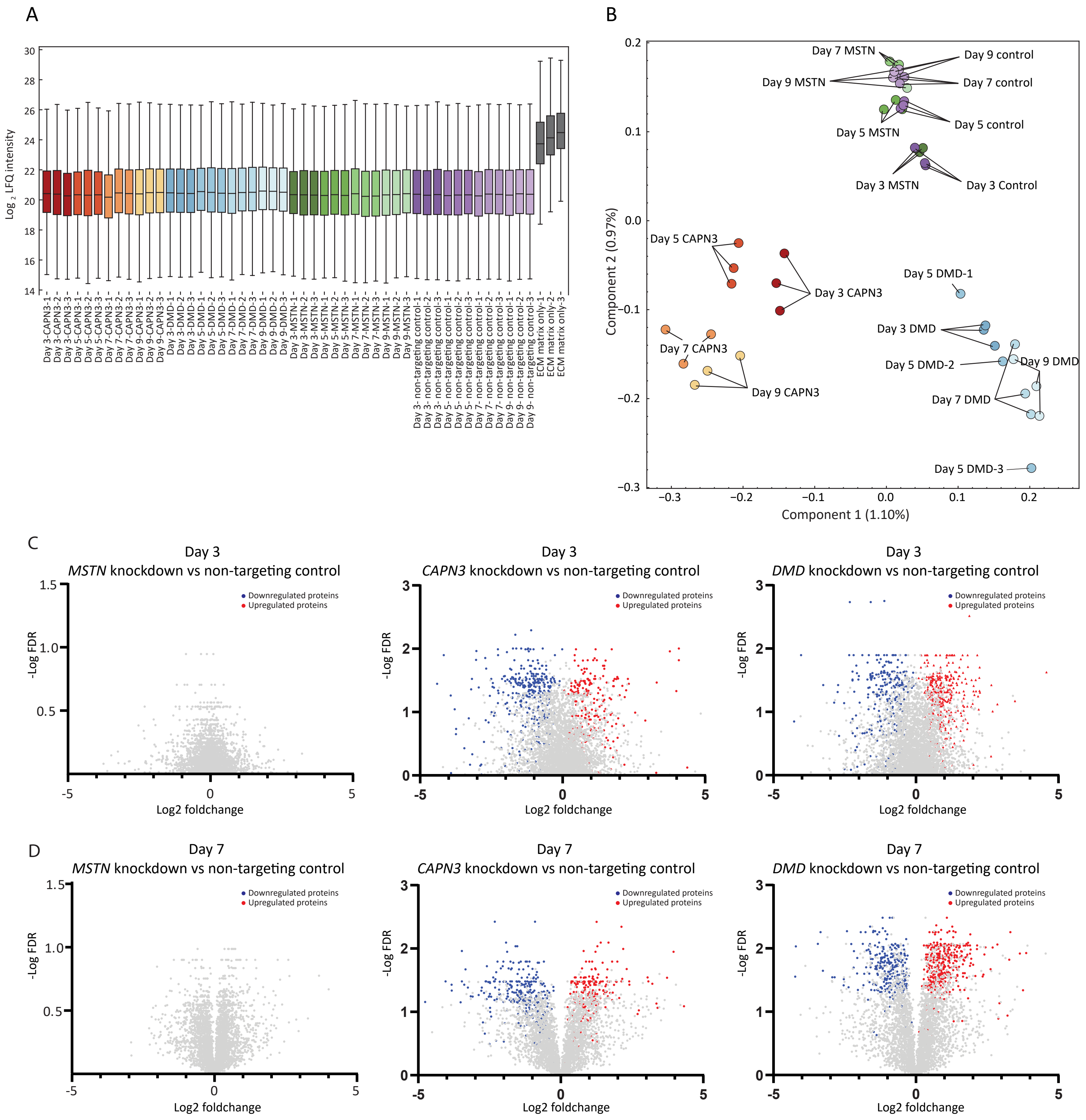

Figure S9 Quality control of the proteomic analysis of 3D-tissue-engineered-skeletal-muscles.

Table S1 hiPSC lines used in this study

hiPSC line	Experiment	Origin	Additional information
HPSI0114i-kolf_3	Figures 1, S1	HipSci platform	https://www.cellosaurus.org/CVCL_AE30
LUMCi003-A	Figures S2–4	LUMC hiPSC Core Facility	[1]
LUMC0162iCTRL05	Figures 2-4, and S5-S9	LUMC hiPSC Core Facility	[2]

1. Buijsen RAM, Gardiner SL, Bouma MJ, van der Graaf LM, Boogaard MW, Pepers BA, et al. Generation of 3 spinocerebellar ataxia type 1 (SCA1) patient-derived induced pluripotent stem cell lines LUMCi002-A, B, and C and 2 unaffected sibling control induced pluripotent stem cell lines LUMCi003-A and B. Stem Cell Res [Internet]. 2018 [cited 2023 Jun 12];29:125–8. Available from: https://pubmed.ncbi.nlm.nih.gov/29656178/

2. van der Wal E, den Hamer B, van der Vliet PJ, Tok M, Brands T, Eussen B, et al. Generation of genetically matched hiPSC lines from two mosaic facioscapulohumeral dystrophy type 1 patients. Stem Cell Res [Internet]. 2019 [cited 2023 Jun 12];40. Available from: https://pubmed.ncbi.nlm.nih.gov/31518905/

Table S2 primer list

Primer	Sequence
LV_integration_Fw	5'-CTGGAAGGGCTAATTCACTC-3'
LV_integration_Rv	5'-GGTTTCCCTTTCGCTTTCAG-3'
human_*ALB*_Fw	5'-GCTGTCATCTCTTGTGGGCTG-3'
human_*ALB*_Rv	5'-ACTCATGGGAGCTGCTGGTTC-3'
RT-qPCR_*GUS*_fw	5'-CTCATTTGGAATTTTGCCGATT-3'
RT-qPCR_*GUS*_rv	5'-CCGAGTGAAGATCCCCTTTTTA-3'
RT-qPCR_*MSTN*_shRNA #1_Fw	5'-GGAGAAGATGGGCTGAATCC-3'
RT-qPCR_*MSTN*_shRNA #1_Rv	5'-TGCTCATCACAGTCAAGACC-3'
RT-qPCR_*MSTN*_shRNA #2_Fw	5'-TACCATGCCTACAGAGTCTG-3'
RT-qPCR_*MSTN*_shRNA #2_Rv	5'-TGTTGTAGGAGTCTCGACGG-3'
RT-qPCR_*CAPN3*_shRNA #1_Fw	5'-ACAGTGTCTGTGAACGAGG-3'
RT-qPCR_*CAPN3*_shRNA #1_Rv	5'-GAGCTTCAGACGGTACTGA-3'
RT-qPCR_*CAPN3*_shRNA #2_Fw	5'-CGCAATTTTCCTATTATCGG-3'
RT-qPCR_*CAPN3*_shRNA #2_Rv	5'-AATCGGGGATTCTCGCAAA-3'
RT-qPCR_*CAPN3*_shRNA #3_Fw	5'-TTGATCGGTCATGCCTAGC-3'
RT-qPCR_*CAPN3*_shRNA #3_Rv	5'-CACAAGGAGGTGCTGAGT-3'
RT-qPCR_*DMD*_shRNA #1_Fw	5'-AGACTGGATGAGACTGGAA-3'
RT-qPCR_*DMD*_shRNA #1_Rv	5'-TTAGACTCCTGTACCTGAA-3'
RT-qPCR_*DMD*_shRNA #2_Fw	5'-CCTAATGAGAGCATAGATG-3'
RT-qPCR_*DMD*_shRNA #2_Rv	5'-TCCTCACTCTCTAAGGAAA-3'
RT-qPCR_*DMD*_shRNA #3_Fw	5'-AGACTCAAACAACTTGCTG-3'
RT-qPCR_*DMD*_shRNA #3_Rv	5'-GCTCAAGAGATCCAAGCAA-3'
RT-qPCR_*DMD*_shRNA #4_Fw	5'-CACAAGCACAGGGTTAGAGG-3'
RT-qPCR_*DMD*_shRNA #4_Rv	5'-CCTACATTGTGTCCTCTCTC-3'
RT-qPCR_*micro-dystrophin*_Fw	5'-GTACATCACATCACTCTTCC-3'
RT-qPCR_*micro-dystrophin*_Rv	5'-GCTAGACTGACCGTGATCTG-3'

Supplemental figure legends

Figure S1 Effect of lentiviral transduction on fusion index of Myogenic Progenitor Cells in 2D.

A. Experimental approach B. Representative images of Myogenic Progenitor Cells (MPCs) transduced with LV.H2B-GFP (method 1, see Figure 1A) at day 3 of differentiation. Red: anti-myosin heavy chain (MHC) antibody. Green: GFP. Blue: nuclei stained with Hoechst. C. Quantification of targeting efficiency and fusion index based on three independent cultures.

Figure S2 Validation of shRNA-mediated knock down of *CAPN3* in 3D-tissue-engineered-skeletal-muscle.

A. Effect of three different shRNAs targeting *CAPN3* mRNA on force-generating capacity in response to twitch or tetanic stimulation of the 3D-TESMs after 7 days of myogenesis. Data are derived from six independent 3D-TESMs and are represented as mean ± SD. shRNA *CAPN3* #1 was used in Figures 2-3. B. RT-qPCR analysis of *CAPN3* expression to determine knock down efficiency. Specific primer sets were designed for each knock down to include the shRNA target sequence within the amplified product. Data are derived from three independent samples (each consisting of two 3D-TESMs) and are represented as mean ± SD. C. Whole mount immunofluorescent staining of A. Green: anti-titin antibody. Blue: nuclei stained with Hoechst. For A and B, statistical significance is indicated for each condition relative to the non-targeting control * = $p < 0.05$, ** = $p < 0.01$, *** = $p < 0.001$.

Figure S3 Validation of shRNA-mediated knock down of *DMD* in 3D-tissue-engineered-skeletal-muscle.

A. Effect of three different shRNAs targeting *DMD* mRNA on force generating capacity in response to twitch or tetanic stimulation of the 3D-TESMs after 7 days of myogenesis. shRNA *DMD* #1 was used in Figures 2-3. Data are derived from five-six independent 3D-TESMs and are represented as mean ± SD. B. RT-qPCR analysis of *DMD* expression to determine knock down efficiency. Specific primer sets were designed for each construct to include the shRNA target sequence within the amplified product. Data are derived from three independent samples (each consisting of two 3D-TESMs) and are represented as mean ± SD. C. Whole mount immunofluorescent staining of A. Green: anti-titin antibody. Blue: nuclei stained with Hoechst. For A and B, statistical significance is indicated for each condition relative to the non-targeting control * = $p < 0.05$, ** = $p < 0.01$, *** = $p < 0.001$.

Figure S4 Validation of shRNA-mediated knock down of *MSTN* in 3D-tissue-engineered-skeletal-muscle.

A. Effect of two different shRNAs targeting *MSTN* mRNA on force generating capacity in response to twitch or tetanic stimulation of the 3D-TESMs after 7 days of myogenesis. shRNA *MSTN* #1 was used in Figures 2-3. Data are derived from five-six independent 3D-TESMs and are represented as mean ± SD. B. RT-qPCR analysis of *MSTN* expression to determine knock down efficiency. Specific primer sets were designed for each construct to include the shRNA target sequence within the amplified product. Data are derived from three independent samples (each consisting of two 3D-TESMs) and are represented as mean ± SD. C. Whole mount immunofluorescent staining of A. Green: anti-titin antibody. Blue: nuclei stained with Hoechst. For A and B, statistical significance is indicated for each condition relative to the non-targeting control * = $p < 0.05$, ** = $p < 0.01$, *** = $p < 0.001$.

Figure S5 Effect of shRNA-mediated knock down on fusion index of Myogenic Progenitor Cells.

A. Experimental approach. The best performing shRNA constructs for each target gene were used (DMD#3, CAPN3 #3, MSTN #3. B. Vector Copy Number analyses of MPCs 48 hours after transduction (n = 3 biological replicates) C. Quantification of the fusion index of MPCs following transduction of the shRNA lentiviral constructs (n = 3 biological replicates, 9 fields per image quantified for each construct). D. Representative images of transduced MPCs at day 4 of differentiation. Green: anti-myosin heavy chain (MHC) antibody. Blue: nuclei stained with Hoechst.

Figure S6 Diameter and alignment of myofibers in the 3D-tissue-engineered-skeletal-muscles analyzed in Figures 2 and 3.

A. Diameter of the myofibers in 3D-TESM tissues used for Figure 2 (n = 40-60 myofibers for each condition). B. Alignment of the myofibers in 3D-TESM tissues used for Figure 2. Myofiber alignment was measured using Δ of the axis (n = 40- 60 fibers per condition, measured in degrees (°)) of each myofiber relative to the average axis of all myofibers. Statistical significance is indicated for each condition relative to the non-targeting control. For B significance was calculated using the distribution of the variance. ns = not significant. *** = $p < 0.001$, **** = $p <0.0001$. # indicates the conditions where the analyses were prevented by the poor quality of the 3D-TESM tissues.

Figure S7 Bright field microscopy of 3D-tissue-engineered-skeletal-muscles in culture.

Bright field microscopy of 3D-TESMs in culture before electrical stimulation. Representative images are shown for the experimental conditions used in Figures 2 and 3 at all four timepoints.

Figure S8 Proteome assessment of myogenesis in 3D-tissue-engineered-skeletal-muscle.

A. Supervised clustering of 3D-TESMs transduced with non-targeting control shRNA, based on the Z-scores of the log 2 LFQ values for all proteins that were found significantly altered between different timepoints. Five clusters with similar expression changes relative to the average LFQ values at all timepoints are indicated. B. Z-scores of the protein clusters shown in A. The results are shown in 2 separate graphs for clarity. C. Top 5 enriched GO pathways in the upregulated protein cluster (cluster 1). D. Top 5 enriched GO pathways in the downregulated protein clusters (cluster 5). E. linearized LFQ values of the major myosin heavy chain isoforms associated with skeletal muscle, represented as the average from three independent 3D-TESMs for all timepoints. F. Heatmap of proteins associated with the development and function of skeletal muscle tissue in 3D-TESMs transduced with non-targeting control shRNAs. Colors and numerical values represent the mean LFQ as a percentage of the most enriched condition.

Figure S9 Quality control of the proteomic analysis of 3D-tissue-engineered-skeletal-muscles.

A. Total protein LFQ values for all protein samples, including the ECM matrix only controls. n = 4715 proteins. B. Principle component analysis of all knock down and control samples. C. Volcano plot of knock downs compared to the non-targeting control at day 3. Proteins that were significantly up and downregulated are indicated in red and blue, respectively. Average protein LFQ values were derived from 3 independent 3D-TESMs from each timepoint. Proteins were considered significant when FDR was $< 0,05$ in ≥ 2 timepoints. D. As C, but now at day 7.

Supplemental methods

Proteomic analysis

Three 3D-TESMs were used per condition on days 3, 5, 7, or day 9 of myogenic differentiation. As a negative control, three cell-free tissues containing only fibrinogen, thrombin, and Matrigel were snap-frozen after 30 minutes of polymerization. Tissues were lysed in 4% SDS, heated to 95°C for 5 minutes, and sonicated for 10 minutes. Protein concentrations were determined using Pierce assay (ThermoFisher), thereafter 10µg protein was reduced and alkylated with 5mM tris (2-carboxyethyl)phosphine (TCEP) and 15mM chloroacetamide (CAA) at 70°C for 10 minutes. Samples were thereafter desalted and washed using SP3 magnetic beads (SpeedBead Magnetic Carboxylate, cytiva, 65152105050250 + 45152105050250). Acetonitrile (ACN) was added to a final concentration of 50% to each sample and incubated for 10 min at room temperature. The samples were placed on a magnetic rack where they were washed twice with 70% Ethanol, once with 100% ACN, air dried, and resuspended in digestion buffer (50mM TEAB with 0.1µg LysC and 0.1µg Trypsin/sample) for overnight incubation at 37°C. Peptides were resuspended in 0.1% formic acid (FA) and purified using Stop and GO extraction tips (StageTips, containing two layers of styrene divinylbenzene-reverse phase sulfonate (SDB-RPS, Affinisep).

Proteome samples were analyzed using liquid chromatography-tandem mass spectrometry on a Q-Exactive™ Plus Hybrid Quadrupole-Orbitrap™ Mass Spectrometer (ThermoFisher). Chromatographic peptide separation was achieved on a 30 cm reversed phase nanoHPLC-columns (150µm inside diameter, and 1.9-µm ReproSil-Pur C18 beads; Dr. Maisch, Germany) coupled to an EASY-nLC 1000 HPLC system and a binary buffer system A (0.1% FA) and B (80% ACN/0.1% FA). Samples derived from SP3 digestion were measured in data-independent acquisition mode (DIA) over a 90 min gradient. Full MS spectra (385–1,015 m/z) were recorded at a resolution (R) of 35,000, maximum injection time (max. IT) of 60 ms and AGC target of 1e6. The isolation window size was set to 24 m/z with staggered windows (12 m/z). HCD fragmentation was performed at nominal collisional energy (NCE) of 27. MS2 spectra were recorded at R = 17,500, a max. IT of 60 ms, and an AGC target of 1e6.

Statistics

Demultiplexing of the generated MS raw data was achieved by ProteoWizard (3.0.21218) with a mass error of 10 ppm. Resulting mzML files were analyzed using the DIA-NN analysis software (1.8.1). Peptides and proteins were identified using the canonical human UniProt database (downloaded 02/2021) with common contaminants and this FASTA was used for a library-free search. Mass ranges were set according to the settings of the MS method, mass deviation was automatically determined from the first data file.

Protein names were used from FASTA, double-pass mode was selected and robust LC with high accuracy was used as quantification strategy. All other parameters were set to the default values.

Obtained results from DIA-NN were further processed in R using an in-house script. The data was filtered for q-value <0.01, Lib.Q.Value <0.01, PG.Q.Value <0.01, Global.Q.Value <0.01, Quantity.Quality >0.7, and Fragment.count ≥4.

Subsequent statistical analysis was performed using Perseus (1.6.15.0). PG.MaxLFQ values were log2 transformed and proteins with less than two peptides were excluded. Raw files were assigned to groups based on the downregulated protein and the isolation timepoint and protein groups were filtered for three valid values in at least one group before missing values were replaced from normal distribution (width: 0.3; down shift: 1.3).

Data were normalized by column-wise median subtraction and two-sided t-tests were performed with a benjamini-hochberg-fdr-correction. FDR cutoff was set to ≤0.05 in ≥ 2 timepoints compared to the respective control, and protein clusters were annotated on the Z-scores of each protein at all four timepoints. Finally, GO pathway enrichment analysis was performed using the protein string database for all protein clusters. Excluding GO pathways comprised of <10 or >250 proteins, and combining pathways of >85% similarity. Graphical visualization was done in Graphpad Prism and Instant Clue (Nolte et al., 2018).

The mass spectrometry proteomics data have been deposited to the ProteomeXchange Consortium via the PRIDE partner repository with the dataset identifier PXD042227.
